# Loss of *swiss cheese* in Neurons Contributes to Neurodegeneration with Mitochondria Abnormalities, Reactive Oxygen Species Acceleration and Accumulation of Lipid Droplets in *Drosophila* Brain

**DOI:** 10.3390/ijms22158275

**Published:** 2021-07-31

**Authors:** Pavel A. Melentev, Elena V. Ryabova, Nina V. Surina, Darya R. Zhmujdina, Artem E. Komissarov, Ekaterina A. Ivanova, Natalia P. Boltneva, Galina F. Makhaeva, Mariana I. Sliusarenko, Andriy S. Yatsenko, Iryna I. Mohylyak, Nataliya P. Matiytsiv, Halyna R. Shcherbata, Svetlana V. Sarantseva

**Affiliations:** 1 Petersburg Nuclear Physics Institute Named by B.P. Konstantinov of NRC «Kurchatov Institute», 188300 Gatchina, Russia; melentev_pa@pnpi.nrcki.ru (P.A.M.); ryabova_ev@pnpi.nrcki.ru (E.V.R.); surina_nv@pnpi.nrcki.ru (N.V.S.); dasha-zhmujdina@yandex.ru (D.R.Z.); komissarov_ae@pnpi.nrcki.ru (A.E.K.); ivanova_ea@pnpi.nrcki.ru (E.A.I.); 2 Institute of Physiologically Active Compounds Russian Academy of Sciences, 142432 Chernogolovka, Russia; boltneva@ipac.ac.ru (N.P.B.); gmakh@ipac.ac.ru (G.F.M.); 3 Institute of Cell Biochemistry, Hannover Medical School, 30625 Hannover, Germany; sliusarenko.mariana@mh-hannover.de (M.I.S.); yatsenko.andriy@mh-hannover.de (A.S.Y.); shcherbata.halyna@mh-hannover.de (H.R.S.); 4 Department of Genetics and Biotechnology, Ivan Franko National University of Lviv, 79005 Lviv, Ukraine; iryna.mohylyak@icm-institute.org (I.I.M.); nataliya.matiytsiv@lnu.edu.ua (N.P.M.)

**Keywords:** hereditary spastic paraplegia, *swiss cheese*, *NTE*, *PNPLA6*, *Drosophila melanogaster*, lipid droplets, mitochondria, neurodegeneration, ROS, oxidative stress

## Abstract

Various neurodegenerative disorders are associated with human NTE/PNPLA6 dysfunction. Mechanisms of neuropathogenesis in these diseases are far from clearly elucidated. Hereditary spastic paraplegia belongs to a type of neurodegeneration associated with NTE/PNLPLA6 and is implicated in neuron death. In this study, we used *Drosophila melanogaster* to investigate the consequences of neuronal knockdown of *swiss cheese* (*sws*)—the evolutionarily conserved ortholog of human *NTE/PNPLA6—*in vivo. Adult flies with the knockdown show longevity decline, locomotor and memory deficits, severe neurodegeneration progression in the brain, reactive oxygen species level acceleration, mitochondria abnormalities and lipid droplet accumulation. Our results suggest that SWS/NTE/PNPLA6 dysfunction in neurons induces oxidative stress and lipid metabolism alterations, involving mitochondria dynamics and lipid droplet turnover in neurodegeneration pathogenesis. We propose that there is a complex mechanism in neurological diseases such as hereditary spastic paraplegia, which includes a stress reaction, engaging mitochondria, lipid droplets and endoplasmic reticulum interplay.

## 1. Introduction

The term “hereditary spastic paraplegia” (HSP) is used to describe a group of heterogeneous neurodegenerative disorders, the predominant feature of which is progressive spasticity of the lower extremities due to axonopathy of corticospinal tract neurons [1]. Additional neurological features of the disease include cognitive impairment, ataxia, dysarthria, neuropathy and seizures [2]. HSP affects individuals of various ethnic groups and ages. The combined prevalence of the disease is estimated to be 1.2–9.6 cases per 100,000 individuals worldwide [3]. HSP is one of the most genetically diverse neurological disorders: there are more than 79 identified genes involved in the development of this disease [2]. Mutations in these genes lead to disruptions of various cellular processes: organelle biogenesis, axon transport, lipid metabolism, DNA repair, myelination, growth of nerve cell processes, vesicular transport, intracellular signaling, mitochondrial function [4,5].

Mutations in the *NTE/PNPLA6* (neuropathy target esterase/patatin-like phospholipase domain containing 6) gene are responsible not only for autosomal recessive HSP (SPG39) [6], but also for other rare neurological syndromes: ataxia with spasticity or pure cerebellar ataxia [7,8,9], Gordon Holmes syndrome, Boucher–Neuhäuser syndrome, Laurence–Moon syndrome, Oliver–McFarlane syndrome and Leber congenital amaurosis [10,11,12,13,14]; also see [15] for review. Neurotoxic organophosphate poisoning results in inhibition of the NTE/PNPLA6 protein and induces the OPIDN (organophosphorus compound-induced delayed neurotoxicity) syndrome [16,17]. *NTE/PNPLA6* orthologs in humans, mice, fruit flies and yeast encode a phospholipase B, which deacylates (lyso)phosphatidylcholine to glycerophosphocholine and (one)/two molecules of free fatty acids [18,19,20,21]. It has been shown that *NTE/PNPLA6* in mice is expressed ubiquitously in the central nervous system, but with age, it is clearly detected only in Purkinje cells, granule cells and pyramidal neurons of the hippocampus and some large neurons in the medulla oblongata, nucleus dentatus and pons [22]. *NTE/PNPLA6* knockout in mice leads to the death of embryos on day 9 of development due to placental defects. Individuals carrying only one normal *NTE/PNPLA6* allele are viable and develop normally, although they have 50% reduced esterase activity [23]. Neuronal deletion of *NTE/PNPLA6* in mice leads to the death of hippocampus and thalamus neurons, as well as cerebellar Purkinje cells [24]. The *NTE/PNPLA6* gene is conservative and its orthologs have been found in prokaryotes and eukaryotes. The similarity of the sequences of gene products is 96% when the mouse and the human are compared, and 36% in comparison between the mouse and the fruit fly [22].

The *NTE/PNPLA6* gene ortholog in *Drosophila melanogaster* is *swiss cheese* (*sws*). SWS protein acts as a phospholipase and as a non-canonical regulatory subunit of a protein kinase A [21,25,26,27]. SWS and the human NTE/PNPLA6 protein have structural (57–61%) and functional homology within their catalytic domains [21,28,29]. Both insect and mammalian proteins are widely expressed in the nervous system [21,22] and are localized in the endoplasmic reticulum of neurons [21,24]. *sws* mutants show apoptotic death of neurons and glia in the fly brain, a shortage of imago life expectancy and disturbances of locomotor behavior [29,30]. Moreover, at the early stages of development, namely in the late pupae, glial processes form abnormal, multilayered wrappings around neurons and axons [30]. SWS function is shown to be important for both neurons and glia [21]. Glia-specific *sws* knockdown induces morphological changes in the fly brain and peripheral nerves, causing locomotion defects [31,32]. At the same time, conditional knockout of *NTE/PNPLA6* in peripheral glia of mice reduces axon wrapping by Schwann cells in the peripheral nerves [33]. However, neuron-specific alterations under *sws* dysfunction in neurons have not been identified yet.

*Drosophila melanogaster* is a powerful model system to study genes that are important for the functioning of the nervous system under both normal and pathological conditions. In this study, we investigate the consequences of neuronal knockdown of the *sws* gene, suggesting this approach to be a model of neuron disturbances accompanying *NTE/PNPLA6*-associated disorders in humans.

## 2. Results

### 2.1. Knockdown of sws in Neurons Induces Neurodegeneration, Longevity Decline and Behavior Deficits

**Figure 1:**
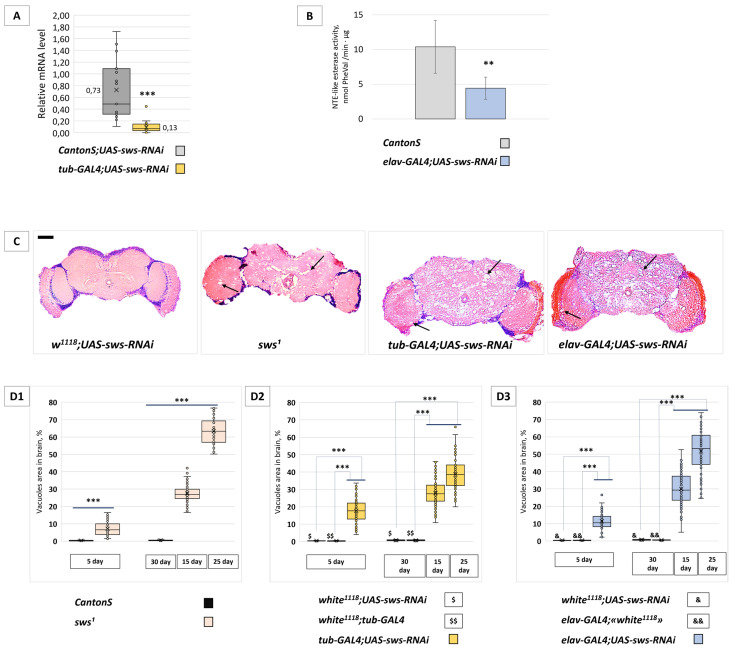
The level of *sws* mRNA, SWS esterase activity and the brain morphology analysis in the case of the RNAi-induced *sws* knockdown. (**A**) Relative *sws* mRNA level in heads of flies with the ubiquitous *sws* knockdown genotype (*tub-GAL4;UAS-sws-RNAi*) and with the control genotype (F1 males obtained from crossing a *CantonS* female and *UAS-sws-RNAi* male). The level of *Gapdh2* mRNA is taken as 1.00 in each sample. The mean value for each genotype is shown. Mann–Whitney test, *** *p* < 0.001, *N* = 18. (**B**) NTE-like esterase activity of fly head lysates from the neuronal *sws* knockdown genotype (*elav-GAL4;UAS-sws-RNAi*) and control genotype (*CantonS*). The mean ± 95% CI, Student *t*-test, ** *p* < 0.01, *N* = 4. (**C**) Hematoxylin and eosin-stained paraffin brain sections of the 25-day-old control flies (F1 males obtained from crossing a *w^1118^* female and *UAS-sws-RNAi* male), *sws^1^* mutants, ubiquitous (*tub-GAL4;UAS-sws-RNAi*) or neuronal (*elav-GAL4;UAS-sws-RNAi*) *sws* knockdowns. Vacuoles in the brain tissue are marked with arrows. Scale bar: 200 μm. (**D1**–**D3**) The total hole area level in the brains of (**D1**) *sws^1^* mutant, (**D2**) the ubiquitous *sws* knockdown (*tub-GAL4;UAS-sws-RNAi*) and (**D3**) the neuronal *sws* knockdown (*elav-GAL4;UAS-sws-RNAi*) flies of different age (coloured boxes). Controls are *CantonS* males (in **D1**); F1 males obtained from crossing a *w^1118^* female and *UAS-sws-RNAi* male ($ and & in **D2**,**D3**); F1 males obtained from crossing a *w^1118^* female and *tub-GAL4* male ($$ in **D2**); F1 males obtained from crossing an *elav-GAL4* female and *w^1118^* male (&& in **D3**). Mann–Whitney (**D1**) or Steel (**D2**,**D3**) test, *** *p* < 0.001, *N* = 82.

Our aim was to analyze specific consequences of *sws* knockdown in neurons. For this purpose, we used transgenic *UAS-sws-RNAi* (BDSC 61338) expression directed against *sws* mRNA in the GAL4/UAS system [34]. First, we evaluated *sws* expression levels in the fly brains of *tub-GAL4;UAS-sws-RNAi* transgenic flies (with ubiquitous *sws* knockdown). RT-qPCR experiments confirmed that *UAS-sws-RNAi* expression resulted in a significant reduction in *sws* mRNA levels (Figure 1A). In the rest of the experiments, we used the *elav-GAL4* transgene for the neuronal *sws* knockdown. We confirmed the effect of *UAS-sws-RNAi* expression at the protein level, showing that SWS esterase activity in the fly brains of the *elav-GAL4;UAS-sws-RNAi* genotype is downregulated (Figure 1B).

**Figure 2:**
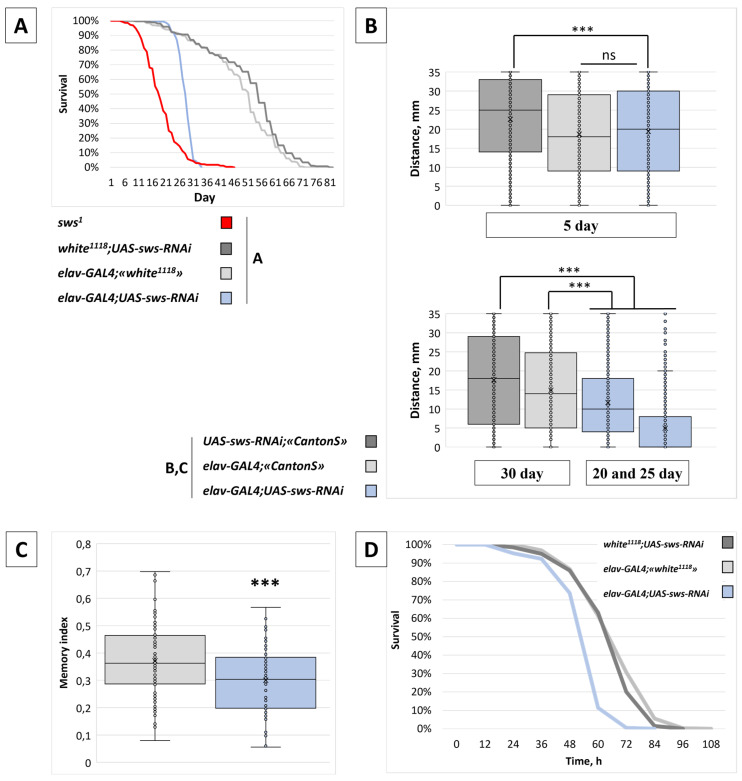
Behavior and survival analysis of flies with the neuronal *sws* knockdown. (**A**) Survival curves for flies with the neuronal *sws* knockdown (*elav-GAL4;UAS-sws-RNAi*, blue line, *N* = 530) *sws^1^* mutants (red line, *N* = 718) and corresponding controls (light grey line for F1 males obtained from crossing an *elav-GAL4* female and *w^1118^* male, *N* = 268; dark grey line for F1 males obtained from crossing a *w^1118^* female and *UAS-sws-RNAi* male, *N* = 234). (**B**) Distance covered by a fly for 3 s after flipping in the RING assay for flies with the neuronal *sws* knockdown (*elav-GAL4;UAS-sws-RNAi*, blue boxes) and for corresponding controls (light grey boxes—F1 males obtained from crossing an *elav-GAL4* female and *CantonS* male; dark grey boxes—F1 males obtained from crossing a *CantonS* female and *UAS-sws-RNAi* male) at different ages. For 20- and 25-day-old knockdowns, we performed a comparison with 30-day-old controls because the performance of the latter was still higher than in the former. Steel test, *** *p* < 0.001, ns—no significant difference (*p* > 0.05), *N* = 2000. (**C**) The memory index for flies with the neuronal *sws* knockdown (*elav-GAL4;UAS-sws-RNAi*, blue boxes) and for corresponding controls (F1 males obtained from crossing an *elav-GAL4* female and *CantonS* male) at the 25th day of life. OY axis—the memory index (see Section 4). Mann–Whitney test, *** *p* < 0.001, *N* = 81. (**D**) Survival curves for starved flies with the neuronal *sws* knockdown (*elav-GAL4;UAS-sws-RNAi*, blue line, *N* = 194) and for corresponding controls (light grey line for F1 males obtained from crossing an *elav-GAL4* female and *w^1118^* male, *N* = 326; dark grey line for F1 males obtained from crossing a *w^1118^* female and *UAS-sws-RNAi* male, *N* = 200).

Next, we performed histological analyses of brain sections stained with hematoxylin and eosin on days 5, 15 and 25–30 of the fly’s adult life. In the 5-day-old knockdown (*elav-GAL4;UAS-sws-RNAi*) flies, we observed increased vacuolization throughout the brain neuropile region and the optic lobes when compared with control flies. Upon aging, the neurodegeneration progressively spread, affecting all areas of the brain (Figure 1C). Since *sws^1^* mutants had shown progressive neurodegeneration in the adult nervous system and reduced lifespan [30], we analyzed whether the neuronal knockdown could phenocopy these effects. With the *sws* knockdown in neurons, the level of neurodegeneration increased significantly compared to the age-related control flies (Figure 1(D3)). A similar pattern was observed in flies with *sws* knockdown in all cells of the body (Figure 1(D2)) and in *sws^1^* loss-of-function mutants (Figure 1(D1)). The lifespan of *sws* knockdown flies and *sws^1^* mutants was significantly shortened compared to the control flies. In *elav-GAL4;UAS-sws-RNAi*, the mean survival was 28 days; in *sws^1^*, it was 22 days; in the controls, it was 50 and 53 days (Figure 2A). These data suggest that the severe neurodegenerative and longevity phenotypes observed in *sws^1^* flies predominantly depend on neuronal *sws* dysfunction. We assume that the *sws* role in neurons, but not in glia, is more critical for the fly’s organism (for comparison, see our results for panglial *sws* knockdown [32], where we show that it does not influence longevity and has a much weaker effect on brain neuropile morphology).

An accelerated neurodegeneration rate could lead to behavior abnormalities. Therefore, we tested whether analyzed knockdowns had locomotion and memory defects. We found that locomotor activity and the memory index decreased in the adult flies with the neuronal *sws* knockdown (Figure 2B,C). Taking into account the severity of the observed phenotypes, we suggest that *sws* downregulation in neurons creates enormous stress for adult *Drosophila* organisms. Then, we decided to check if an additional stressor could have even more deleterious effects. To test this hypothesis, we analyzed the survival rate of starved young flies and observed the reduction in the median lifespan for the knockdown (Figure 2D).

Besides this, another *UAS-sws-RNA* transgene (VDRC v5469) was also appropriate to reduce the *sws* mRNA level in the heads of *nSyb-GAL4;UAS-sws-RNAi^v5469^* or *repo-GAL4;UAS-sws-RNAi^v5469^* flies (Figure S1). However, despite promoting the well-characterized glia phenotypes [31], it did not cause any neurodegeneration when expressed under the *elav-GAL4* driver (Figure S1). This could be due to the smaller effect size, which is probably not enough for the neuronal death, while both *UAS-sws-RNAi^v5469^* and *UAS-sws-RNAi* (BDSC 61338) are enough to induce glia abnormalities [31,32]. Thus, we conclude that the *UAS-sws-RNAi^v5469^* transgene is insufficient to induce *sws* knockdown in neurons; therefore, we did not use it in other experiments of the current work.

### 2.2. The sws Gene Is Expressed in Mushroom Body and Is Necessary for Its Normal Morphology

**Figure 3:**
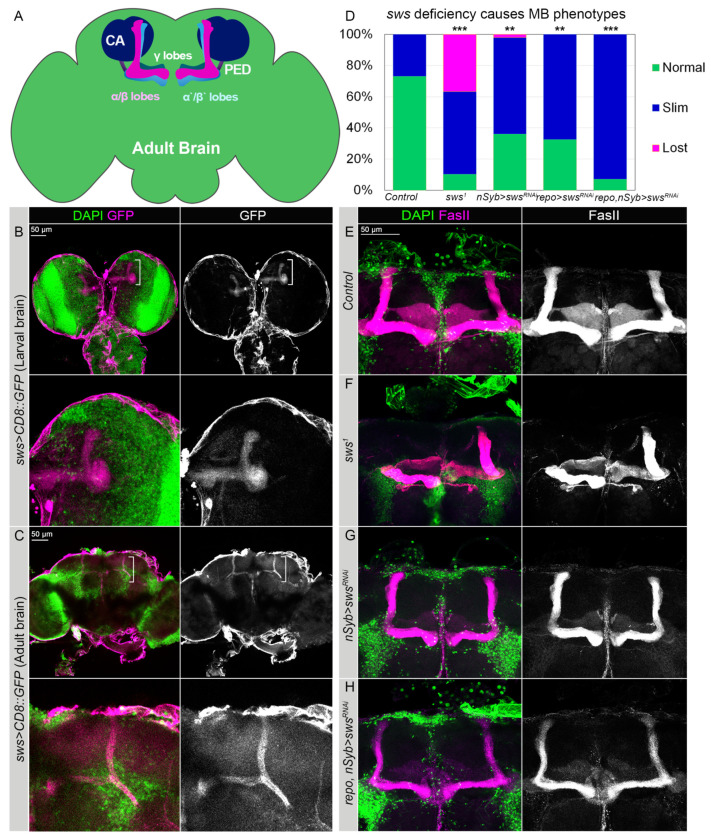
SWS expressed in MB neuropile is required for the proper MB assembly. (**A**) Schematics of the adult *Drosophila* brain showing mushroom body (MB) neuropile. Calyx (CA, night blue), pedunculus (PED, dark violet), α/β (pink), α’/β’ (blue) and γ (tile) lobes. (**B**,**C**) The *sws* promoter activity pattern in the larval (**B**) and adult (**C**) brains detected by GFP expression (magenta, *UAS-CD8::GFP*) under control of the *sws-GAL4* driver. Note the *sws* expression pattern in the MB marked with white parentheses. Enlarged MBs are shown in the panels below. (**D**) Frequency of the appearance of abnormal MB lobes in the *sws* dysfunction genotypes. Two-way tables and chi-square test was used for statistical analysis, ** *p* > 0.005, *** *p* > 0.001, *N* > 25. (**E**–**H**) Confocal 3D-images of adult brains stained with FasII antibodies (magenta). Comparison of the control (**E**) and the *sws^1^* loss-of-function mutant males (**F**) shows that α/β MB lobes are disorganized in the latter. (**G**) Upon neuronal *sws* downregulation (*nSyb-GAL4;UAS-sws-RNAi)*, α/β MB lobes appear underdeveloped. (**H**) This phenotype is even more pronounced when *sws* is downregulated in both neurons and glia (*repo-GAL4;nSyb-GAL4;UAS-sws-RNAi).* Fifteen-day-old males were analyzed. DAPI (green) marks nuclei. Scale bars: 50 µm.

Similar to the human brain, the *Drosophila* brain is compartmentalized. Different compartments have highly specific functions and are responsible for the control of different behaviors. One of the most prominent compartments is the mushroom body (MB) neuropile, which is involved in learning, memory storage and control of the sense of smell and startle-induced locomotion [35,36,37,38,39,40,41]. Intrinsic neurons of MB, or Kenyon cells, arborize their dendrites in calyces, while their axons grow in a bundle called a pedunculus, at the end of which they separate and form vertical α/β, α’/β’ and medial γ lobes (Figure 3A).

We found the *sws* promoter to be active in the MB lobes of larval and adult fly brains (Figure 3B,C). Importantly, our results show that SWS is required for the proper development of MB lobes. In particular, in *sws^1^* mutants, the late-born α/β lobes were significantly underdeveloped and showed axon pathfinding phenotypes (Figure 3F). This phenotype partially depends on the neuronal SWS function. Upon *sws* knockdown, using the neuronal *nSyb-GAL4* driver, which has high expression levels in preadult as well as in adult fly brains, MB lobes appeared significantly underdeveloped, which was similar to the *sws*^1^ mutants’ brains (Figure 3G).

However, the frequency of abnormal MB phenotypes under *sws* knockdown in neurons (approximately 65%) was lower than in *sws^1^* loss-of-function mutants (approximately 90%*,* Figure 3D). The results suggest that the SWS deregulation in glia could additionally contribute to the observed anomalies in the MB organization. To test this hypothesis, we downregulated *sws* specifically in the glia using *repo-GAL4* driver, which also resulted in the appearance of brains with abnormal MB lobes (Figure S2). Upon *sws* knockdown in both neurons and glia, the frequency of abnormal MB lobes was similar to the *sws^1^* loss-of-function mutants (>90%, Figure 3D,H). These findings suggest that the normal presence of SWS in both neurons and glia is important for the proper development of the MB neuropile. This also means that not only neurons but also glia are essential for MB neuropile development in adult flies, which was stated previously [42].

### 2.3. Knockdown of sws in Neurons Induces Various Molecular Events in the Organism

To reveal regulation pathways that are activated or repressed in the organism in the case of *sws* knockdown, we analyzed a whole-fly transcriptome, using a microarray approach. The rationale for this is multiple functions that are under the control of *sws* expression. Dysfunction of *sws* in neurons could alter protein kinase A activity (since SWS acts as a regulatory subunit of protein kinase A), intracellular lipid metabolism regulation pathways (since SWS is a lysophospholipase), intercellular interactions (since neurons operates a whole organism) and adaptive reactions (since degeneration of the nervous tissue would cause inflammation, for instance). In 25-day-old males with neuronal *sws* knockdown, we found 940 genes that were downregulated in comparison to the wild-type *CantonS* males. These genes included those which regulate male gamete generation, microtubule organization, transport of dicarboxylic acids that take part in gluconeogenesis, the urea cycle, the glyoxylate cycle, amino acid synthesis, fatty acid synthesis and the citric acid cycle. Among the 589 genes that were upregulated in the organism of the 25-day-old knockdowns, the most notable overrepresented processes were: phototransduction and visual perception, metabolism of amino acids, carboxylic acids and lipids, defense response and GPCR signaling. Additionally, there were a number of upregulated genes (77, to be exact) that ordinarily participate in various oxidation-reduction processes; some of them (12, to be exact) control the major intracellular antioxidant system, which implies their involvement in glutathione metabolism (Figures S3–S6).

We propose then that not only the transcriptomic but also proteomic changes can be observed in response to *sws* downregulation. We found that in the heads of males with the *sws^1^* mutation, distinct proteomic alterations occurred in comparison with wild-type *CantonS* males (Data S2 and S3). Among the identified differentially expressed proteins, there were some that corresponded to the genes up/downregulated in the organism of flies with the neuronal *sws* knockdown. For instance, the levels of the antioxidant defense proteins GSTD2, GSTE7 and MGSTL were two or more times higher in the heads of *sws^1^* males compared to *CantonS* males (and the corresponding genes were upregulated in *elav-GAL4/UAS-sws-RNAi* males compared to *CantonS* males). In addition, a similar correlation was found for the chaperone HSP26 and the lipid metabolism and turnover proteins: ACCCOAS, CG12512, CG1648, CG17597, MTP. Furthermore, 60 of the upregulated proteins and 23 of the downregulated proteins in mutants’ heads were found to be representatives of the lipid droplet (LD) subproteome [43]. As for our transcriptomic results, 34 of the upregulated genes and four of the downregulated genes corresponded to the LD-associated proteins (Data S3).

### 2.4. Knockdown of sws in Neurons Is Accompanied by Oxidative Stress

**Figure 4:**
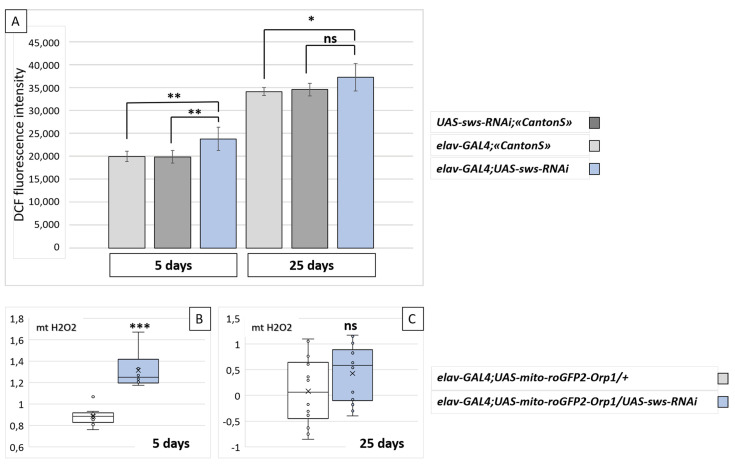
Oxidant levels in the brains of flies with the neuronal *sws* knockdown. (**A**) Fluorescent signal levels corresponding to the total reactive oxygen species concentration in the brain samples of flies with the neuronal *sws* knockdown (*elav-GAL4; UAS-sws-RNAi*, blue boxes) and of corresponding controls (light grey boxes—F1 males obtained from crossing an *elav-GAL4* female and *CantonS* male; dark grey boxes—F1 males obtained from crossing a *CantonS* female and *UAS-sws-RNAi* male) at different ages. Mean ± 95% CI, Dunnett’s test, ** *p* < 0.01; * *p* < 0.05, ns—no significant difference (*p* > 0.05), *N* = 7. (**B**,**C**) Relative level of hydrogen peroxide in neuron mitochondria of the brain in *sws* knockdowns (*elav-GAL4;UAS-mito-roGFP2-Orp1/UAS-sws-RNAi*, blue boxes) and in controls (*elav-GAL4;UAS-mito-roGFP2-Orp1/+*, white boxes) at the 5th (**B**) and the 25th (**C**) day. OY axis—relative ratio of the sensor fluorescence (see Section 4). Mann–Whitney test, *** *p* < 0.001, ns—no significant difference (*p* > 0.05), *N* = 12.

We found the upregulation of antioxidant defense genes in the organism of flies with the neuronal *sws* knockdown, suggesting that oxidative stress may be a potential consequence of *sws* downregulation. Moreover, in our previous study, we found brain ROS acceleration under *sws* knockdown in glia [32]. In addition, it is well known that oxidative stress is a hallmark of neurodegeneration, even though the interrelations between cell death rate and reactive oxygen species accumulation remain elusive [44]. There is evidence that the etiology of many neurodegenerative diseases, including HSP, may involve the generation of reactive oxygen species (ROS), which are associated with mitochondrial dysfunction [45,46]. Therefore, we assessed the ROS level in fly brains, using 2′,7′-dichlorodihydrofluorescein diacetate [47]. As shown in Figure 4A, we found that ROS levels were higher in the brains of the 5-day-old *sws* knockdowns compared to the control flies. This could be partially due to the observed elevation of the mitochondrial H_2_O_2_ levels in the neurons of flies with the *sws* knockdown (Figure 4B). However, in the 25-day-old flies, there was no statistically significant difference between the control and the knockdown genotypes for both the brain ROS and the neuron mitochondria H_2_O_2_ levels (Figure 4C).

### 2.5. Knockdown of sws in Neurons Reduces Mitochondria Signal in Brain and the Number of Mobile Mitochondria in Wing Axons

**Figure 5:**
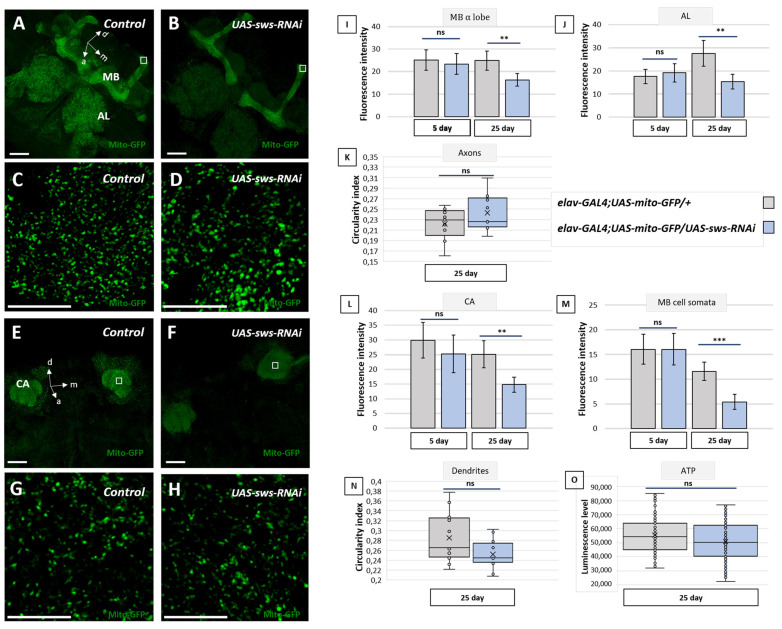
Fly brain mitochondria analysis. (**A**–**H**) Three-dimensional confocal images of the brains of 25-day-old flies of the control (*elav-GAL4;UAS-mito-GFP/+*) and the *sws* knockdown (*elav-GAL4;UAS-mito-GFP/UAS-sws-RNAi*) genotype, GFP (green) is localized in the neuron mitochondria. (**A**–**D**) Mushroom body (MB) and antennal lobes (AL); (**E**–**H**) calyx (CA) and cell bodies. (**A**,**B**,**E**,**F**) Whole view, scale bar: 25 µm; (**C**,**D**,**G**,**H**) magnification of the respective white rectangles, scale bar: 10 µm. (**I**,**J**,**L**,**M**) Fluorescence intensity in the axons of the bulbous tip in the α lobe of the MB (**I**), the antennal lobe (**J**), dendrites (CA) (**L**) and MB cells’ somata (**M**) in brains of 5- and 25-day-old flies of the control (*elav-GAL4;UAS-mito-GFP/+*, grey boxes) and the *sws* knockdown (*elav-GAL4; UAS-mito-GFP/UAS-sws-RNAi*, blue boxes) genotype. Mean ± 95% CI, Student *t*-test, ** *p* < 0.01, ns—no significant difference (*p* > 0.05), *N* = 25. (**K**,**N**). The mitochondrial circularity index for axonal (**K**) and dendritic (**N**) mitochondria of MB cells in the control (*elav-GAL4;UAS-mito-GFP/+*, grey boxes) and *sws* knockdown (*elav-GAL4;UAS-mito-GFP/UAS-sws-RNAi*, blue boxes) 25-day-old flies. A central location from the anterior view was used to characterize dendritic and axonal mitochondria. a—anterior, m—medial, d—dorsal. Mann–Whitney test, *** *p* > 0.001, ns—no significant difference (*p* > 0.05), *N* = 12. (**O**) The total luminescence level corresponding to the ATP concentration in the heads of 25-day-old flies with the *sws* knockdown in neurons (*elav-GAL4;UAS-sws-RNAi*, blue box) and in the control flies (F1 males obtained from crossing an *elav-GAL4* female and *w^1118^* male, grey box). Mann–Whitney test, ns—no significant difference (*p* > 0.05), *N* = 100.

Since our data demonstrate that the levels of ROS are increased upon *sws* deficiency in the brain and it has been shown that elevated ROS levels are often accompanied by abnormalities in the mitochondrial morphology [48,49], we investigated the distribution of mitochondria in the brains of flies with the *sws* knockdown using the *UAS-mito-GFP* transgene (the reporter construct coding GFP fused to a mitochondrial targeting signal [50]). In the control *elav-GAL4;UAS-mito-GFP* flies, the highest signal of mitochondria in the brain was found in the MB, the antennal lobes and the central complex (Figure 5A). In the case of the neuronal *sws* knockdown, the mito-GFP signal was significantly decreased in all these structures by the 25th day of adult life (Figure 5B,I,J,L,M), suggesting the possibility of a global reduction in mitochondria number. It should be mentioned that in young 5-day-old flies, there was no statistically significant difference between the knockdown and the control, suggesting that the decrease in the mitochondria numbers observed in the *sws*-deficient animals is age-dependent.

In addition, we tested whether *sws* knockdown induced any functional damage to the remaining mito-GFP-positive mitochondria in 25-day-old flies. We did not find any alterations in the mitochondria shape in the brains of the neuronal *sws* knockdown flies. Similar to the controls, mitochondria of the knockdowns still had an elliptic shape in both the calyx and pedunculi of an MB (Figure 5K,N). Moreover, the total brain ATP level was not disturbed upon the knockdown (Figure 5O).

**Figure 6:**
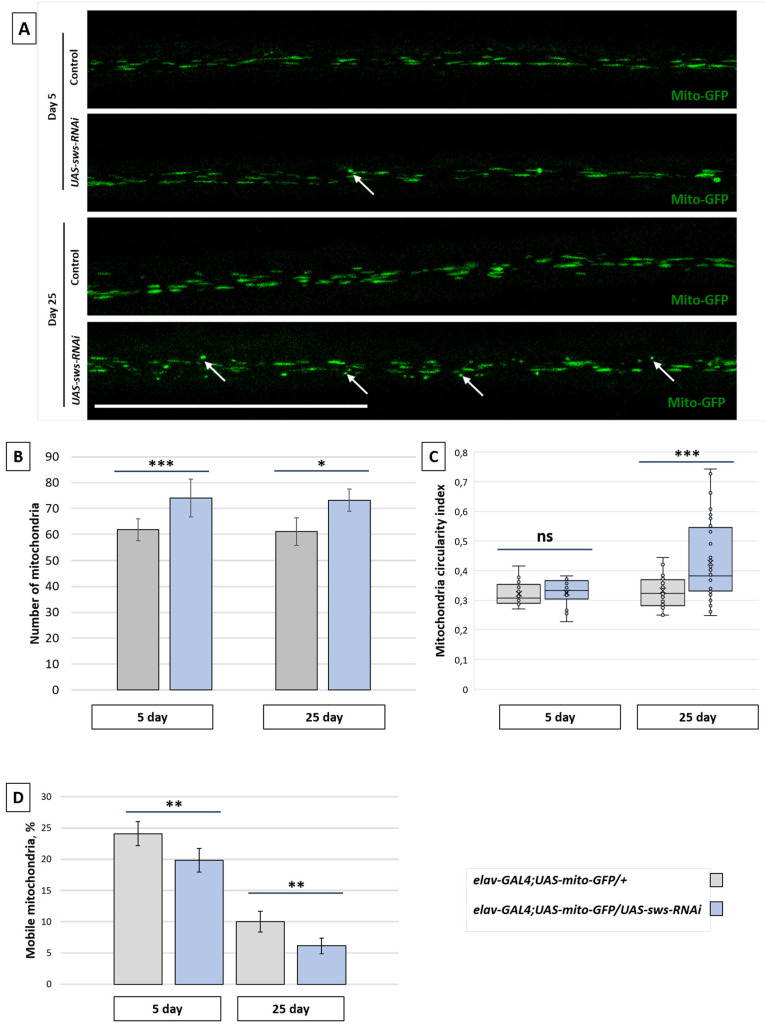
Mitochondria analysis in the fly wing. (**A**) Three-dimensional confocal images of the wing nerves of 25-day-old flies of the control (*elav-GAL4;UAS-mito-GFP/+*) and the *sws* knockdown (*elav-GAL4;UAS-mito-GFP/UAS-sws-RNAi*) genotype, where GFP (green) is localized in the neuron mitochondria. White arrows indicate circular (non-functional) mitochondria. Scale bar: 50 µm. (**B**) The total number of mitochondria in the wing axons of the 5-day-old and 25-day-old control (*elav-GAL4;UAS-mito-GFP/+,* grey boxes) and knockdown (*elav-GAL4;UAS-mito-GFP/UAS-sws-RNAi*, blue boxes) flies. Student *t*-test, *** *p* < 0.001, * *p* < 0.05, *N* = 15. (**C**) The mitochondrial circularity index for the wing axon mitochondria of the 5-day-old and 25-day-old control (*elav-GAL4;UAS-mito-GFP/+,* grey boxes) and knockdown (*elav-GAL4;UAS-mito-GFP/UAS-sws-RNAi*, blue boxes) flies. Mann–Whitney test, *** *p* < 0.001, ns—no significant difference (*p* > 0.05), *N* = 25. (**D**) The percentage of mobile mitochondria in the wing axons of the 5-day-old and 25-day-old control (*elav-GAL4;UAS-mito-GFP/+,* grey boxes) and knockdown (*elav-GAL4;UAS-mito-GFP/UAS-sws-RNAi*, blue boxes) flies. Mean ± 95% CI, Student *t*-test, ** *p* < 0.01, *N* = 25.

In order to analyze mitochondria more precisely, not only in the CNS but also in the PNS, we examined the long wing axons (in the L3 wing vein) of adults. The shape of mitochondria in the 25-day-old knockdown flies was more circular than in the control (Figure 6A,C), corresponding to probably distressed organelles [49]. While the number of mitochondria was increased in the knockdown compared to the control in both 5- and 25-day-old flies (Figure 6B), the percentage of mobile mitochondria was decreased (Figure 6D). This was the case of neither anterograde nor retrograde mitochondria tracking defects, since no change in mitochondrial flux or tracking speed was observed (Figure S7).

### 2.6. Knockdown of sws in Neurons Induces Lipid Droplet Accumulation in Brain

**Figure 7:**
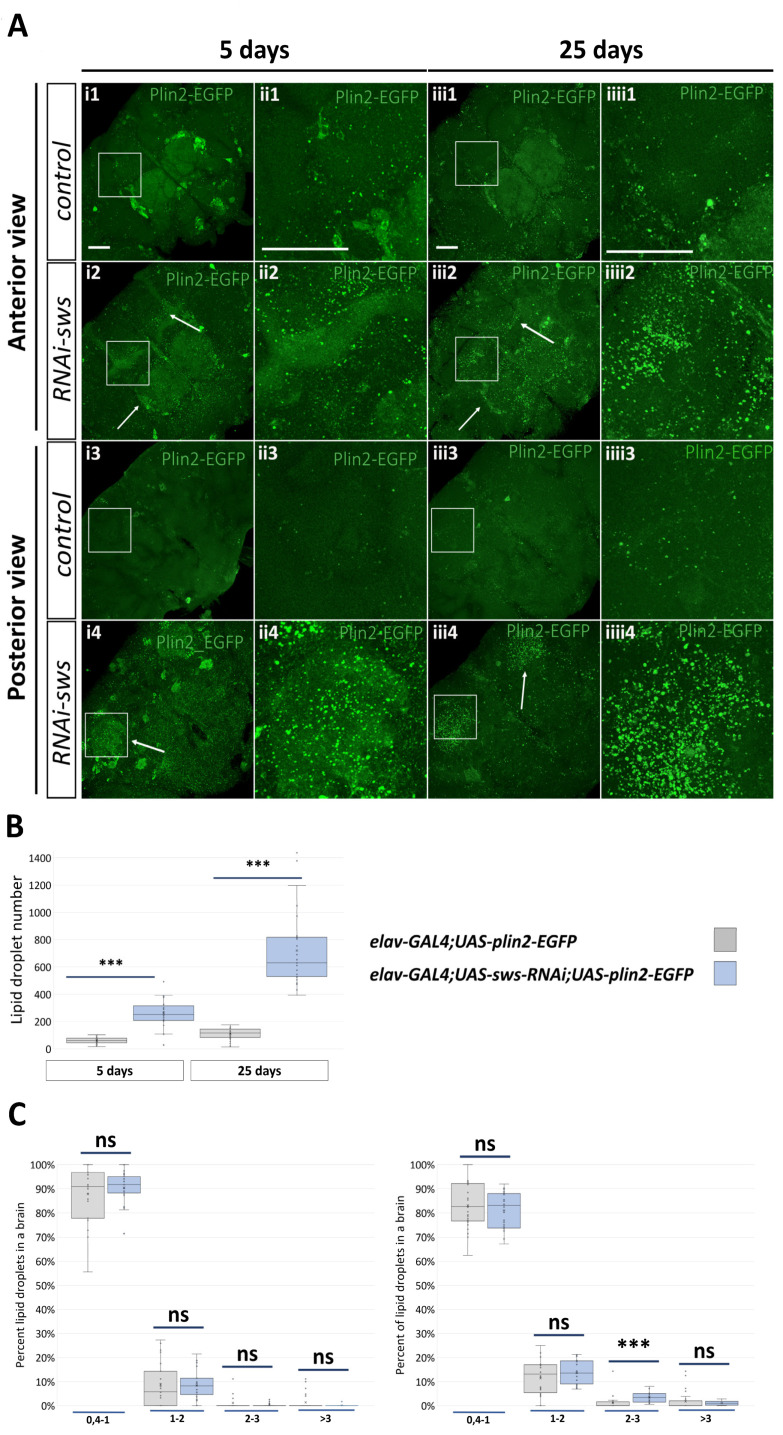
Analysis of lipid droplets in neurons of the fly brain. (**A**) Representative 3D stacks of confocal images of the ventral (**1**,**2**) and the dorsal (**3**,**4**) half of the brain in the control (**1**,**3**, *elav-GAL4;UAS-plin2-EGFP*) and knockdown (**2**,**4**, *elav-GAL4;UAS-sws-RNAi;UAS-plin2-EGFP*) flies aged 5 (**i**,**ii**) and 25 (**iii**,**iiii**) days. Green: GFP embedded in lipid droplets via the Plin2 protein in neurons. Arrows indicate lipid droplets in the mushroom body lobes and antennal lobes (**i2**,**iii2**) and in the calyxes (**i4**,**iii4**). Scale bar: 50 µm. (**B**) The total lipid droplet number in the brain neurons of the control (*elav-GAL4;UAS-plin2-EGFP*, grey boxes) and knockdown (*elav-GAL4;UAS-sws-RNAi;UAS-plin2-EGFP*, blue boxes) flies aged 5 and 25 days. Mann–Whitney test, *** *p* < 0.001, *N* = 29. (**C**) Distribution of LD size (µm^2^) in the brain neurons of the control (*elav-GAL4;UAS-plin2-EGFP*, grey boxes) and knockdown (*elav-GAL4;UAS-sws-RNAi;UAS-plin2-EGFP*, blue boxes) flies aged 5 and 25 days. Mann–Whitney test, *** *p* < 0.001, ns—no significant difference (*p* > 0.05), *N* = 29.

It is well known that LDs accumulate in various cell types under stress conditions, especially in those that are associated with mitochondrial dysfunction and/or ROS acceleration [51,52,53,54,55,56]. In this study, we elucidate mitochondria alterations, ROS acceleration, induction of antioxidant-associated genes expression and LD gene/protein overrepresentation upon *sws* dysfunction. In addition, SWS is known to be a (lyso)phospholipase itself, acting in lipid metabolism [21,57]. Therefore, we decided to test whether LDs could accumulate in the fly brain in the case of the neuronal *sws* knockdown. We analyzed the localization of neuronally expressed EGFP-tagged perilipin 2, the protein specifically located in the LD monolayered membrane [58]. We found that the *sws* knockdown caused a more than four-fold increase in the LD number in the knockdown fly brains on day 5 and 25 (Figure 7A,B). Although we found a small increase in the percentage of 2–3 µm^2^ LDs in the knockdown flies compared to the control flies, we concluded that the total elevation of the LD number was not due to the particular LD size overrepresentation, but rather due to LD augmentation of all possible sizes (Figure 7C,D). Interestingly, in *elav-GAL4;UAS-sws-RNAi;UAS-plin2-EGFP* flies, perilipin 2 was localized predominantly in the MB and the antennal lobes (Figure 7A), i.e., the parts of the brain where we observed the reduction in the mitochondria signal (Figure 5B). In contrast, the panglial *sws* knockdown did not alter the number, the size or the distribution of brain glial LDs (Figure S8).

## 3. Discussion

In this study, we showed that the neuronal knockdown of *sws*, an ortholog of human *NTE/PNPLA6*, recapitulates the key features of motor neuron diseases associated with this gene, namely age-progressive neurodegeneration and locomotor activity decline. In the aged (25-day-old) flies with the *sws* knockdown, the memory index was reduced. In addition, we found a lifespan shortage under normal and stressful conditions. In contrast to the neuronal knockdown, glial-specific *sws* knockdown did not lead to a lifespan reduction [32], but did result in behavioral defects and brain vacuolization, albeit much less intensive [31,32]. Both neuronal and panglial knockdowns led to accelerated ROS levels in the brains of 5-day-old flies. In the case of *elav-GAL4;UAS-sws-RNAi*, the hydrogen peroxide level was also increased in the neuron mitochondria.

Oxidative stress, in fact, is one of the factors associated with the pathogenesis of HSP [46,59,60,61,62] and other neurodegenerative diseases such as Alzheimer’s [63,64,65,66,67,68], Parkinson’s [69,70,71], Huntington’s [72,73,74], amyotrophic lateral sclerosis [75,76,77,78,79,80] and Fredrich’s ataxia [81,82,83,84]. The main sources for ROS in a cell are mitochondria, which produce ROS via the electron transport chain during the process of oxidative phosphorylation [85,86]. Dynamic fusion and fission as well as constant regulation of oxidation-reduction reactions are crucial for mitochondria’s functional stability [87,88]. Disruption of these processes and alterations in mitochondria’s structural and functional stability lead to oxidative stress, mitochondria damage and apoptosis [89,90], e.g., it is shown that HSP-associated genes, when disrupted, also lead to defects in mitochondria, resulting in the increased pathological effects due to accelerated sensitivity to oxidative agents [91,92,93,94]. In addition, HSP-associated genes’ malfunction results in a lack of oxidative phosphorylation [91,95,96], which in turn activates the stress response [97,98] and affects axonal transport [99,100]. At the same time, it is becoming more and more evident that the redox state of cells and mitochondrial homeostasis are closely interrelated, whereas oxidative stress could have a beneficial role [49,101,102].

Under neuronal *sws* knockdown, we detected an abnormal mitochondria signal in the mushroom bodies, both in pedunculi (where Kenyon cells’ axons are located) and the calyx (where Kenyon cells’ dendrites are located). However, no changes in mitochondria morphology and ATP levels were observed, suggesting that, on the one hand, mitochondria are sensitive to the *sws* knockdown, and, on the other hand, at least most of them remain functional. However, this is not enough for the survival of neurons. A completely different situation was observed in the long axons of the wing. Knockdown of *sws* resulted in an increase in the total number of mitochondria. Moreover, we found the escalation of the circularity index of these mitochondria by the 25th day of adult life, indicating that the accumulation of spherical organelles occurred. At the moment, it is difficult to conclude if the observed phenotypes are caused by some changes in the mitochondria dynamics or a disruption of actual organelles. In order to address this, more detailed studies are required. We have not identified any changes in the velocity of axonal transport of mitochondria, while the motile mitochondria number was reduced with the *sws* knockdown. The possible explanation for this could be a general reduction in functional mitochondria, although the total number was increased. We presume that the observed differences in mitochondrial homeostasis in the axons of the MBs and in the wing axons depend on the length of the latter. It is well known that HSP pathogenesis always implies an initial disturbance of long axons [4,103,104,105]. A plethora of experiments shows that the ablation of HSP-related genes infers mitochondrial dynamics in axons, however, sometimes indirectly, through an endoplasmic reticulum state [106,107,108,109,110]. Furthermore, we have previously found SWS to be present presynaptically in larval neuromuscular junctions, and its dysfunction altered their organization, influencing mitochondria distribution [111].

The growing body of evidence elucidates the LDs’ role in cell protection [54,112,113,114,115,116,117,118]. Being cytoplasmic dynamic organelles, LDs store fatty acids, mainly as triglycerides, and supply a cell with fatty acids for energy production, lipid metabolism and membrane turnover. Furthermore, they sequester lipids from β-oxidation in mitochondria or ROS-dependent oxidation in cytoplasm. LDs interact with many cellular organelles, including mitochondria, peroxisomes, lysosomes, the endoplasmic reticulum and the nucleus. In stress conditions, LDs have a supportive role, reducing mitochondrial and endoplasmic reticulum stress, protein or lipid toxicity effects [119,120,121,122,123]. LDs are abundant in various organs in animals, including the gut, fat tissue and the brain in *Drosophila melanogaster* [120]. In the fruit fly nervous system, LDs are found in retinal pigment glia [52,124], cortex and subperineurial glia [54,125], as well as in perineurial and neuropile glia [125] and astrocyte-like epithelial glia [52]. Additionally, a recent work has found LDs in photoreceptor neurons [126]. The amount of LDs in a brain under normal conditions is minimal, in contrast to other tissues of the body [127]. It has been established that mammalian cells (including both neurons and glia) can accumulate lipid droplets in pathological conditions and in modeling various neurodegenerative disorders, such as Huntington’s and Alzheimer’s disease [128,129,130,131,132], as well as HSP [133,134,135,136]. Several HSP-associated proteins have been shown to affect LD dynamics: spartin, spastin, atlastin-1, seipin and REEP1 [135,137,138,139,140,141]. A recent study has shown that the C-terminal region of NTE/PNPLA6, but not the full-length protein, could also be physically attached to LDs with high affinity, inducing the clustering of the latter [142].

In the current study, we have shown that the downregulation of *sws* expression in neurons leads to the accumulation of LDs in these cells. The pattern of LD distribution in flies with the neuronal *sws* knockdown differed from that typically observed in the *Drosophila* adult brains, with very few or even no droplets in neurons [125,143]. While the LD number substantially accelerated in neurons of *elav-GAL4;UAS-sws-RNAi* flies, the *sws* knockdown in glia did not cause any changes in the LD number in glial cells, where droplets were as abundant as in the control genotype. It is important to note that our LD visualization system implied *plin2* overexpression in both control and knockdown genotypes, probably contributing to the excessive stabilization of LDs in neurons, as has been recently shown [126]. Interestingly, abundant LDs have been found in the brains of *DDHD2* knockout mice, accumulating mainly in neurons within their somata, dendrites and axons. Similar to *NTE/PNPLA6*, *DDHD2* encodes a serine hydrolase that exhibits phospholipase A1/TAG hydrolase activity [136,144]. Mutations in both *NTE/PNPLA6* and *DDHD2* lead to HSP [6,145].

The role of LDs in cell survival is controversial and not very clear. It has been shown that LD accumulation alone is insufficient to cause neurodegeneration in *Drosophila*, and ROS is required in conjunction with LD accumulation to promote neurodegeneration [52]. At the same time, the reduction of lactate transport and LD enrichment in glia could alleviate neurodegeneration despite a high ROS level [53]. However, disruption of APOE-dependent lipid transport together with ROS induction decrease LDs and promote neurodegeneration. This fact suggests that glial LD formation and accumulation triggered by elevated levels of ROS provide a protective mechanism against neurodegeneration [53]. Moreover, the protective role of glial LDs is found in the nervous stem cell niche. Suppression of LD formation through the DGAT1 enzyme in glia results in diminished neuroblast proliferation, whereas *plin2* knockdown in glia induces glial LD loss and oxidative stress in neuroblasts [54]. On the contrary, LD accumulation in germline stem cells in *Drosophila* testes is thought to be detrimental for cell survival [55]. In our study, upon *sws* knockdown, we observed substantial LD accumulation in neurons and severe neurodegeneration in the neuropile. Surprisingly, initially increased ROS in the brains of young flies reverted to the normal level in 25-day-old flies with the neuronal *sws* knockdown. This suggests the possibility of LDs’ involvement in neuroprotection, probably via a compensatory response to soften oxidative stress. However, the observed changes in LDs are still not enough to prevent the neurodegeneration. Interestingly, till the 25th day of the imago’s life, we observed LD accumulation in the areas that, in normal conditions, have the highest mitochondria signal, namely in the MBs and the antennal lobes, which are also affected upon *sws* knockdown. MBs are structures in insect brains that regulate memory formation [146], receiving a perception signal from the antennal lobes that process sensory information [147]. The central complex, where we found the pronounced mito-GFP signal too, is required for the maintenance of locomotor activity in flies [148]. Both memory and locomotor behavior were reduced in flies with the neuronal *sws* knockdown.

In summary, our study shows that the knockdown of *sws* in *Drosophila melanogaster* neurons leads to mitochondrial abnormalities, especially in the long wing axons, ROS acceleration and accumulation of LDs in the brains of flies, which is accompanied by neurodegeneration, impaired locomotor activity and longevity decline. Taking into account the (lyso)phospholipase function of the SWS protein and corresponding consequences in lipid metabolism in the case of *sws* dysfunction [21], we propose a SWS/NTE/PNPLA6 role in a wider stress response that implicates both mitochondria and LD lipid dynamics. Recent research shows that mitochondria and LDs can be physically connected to each other [149], and this interaction has many functional features [150]. In addition, the *sws* mutation, comprising a lysophosphatidylcholine increase, induces endoplasmic reticulum stress [151]. Moreover, the endoplasmic reticulum lipid composition is closely related to both mitochondria and LD interrelations [122]. It is noteworthy that the alterations of the abovementioned organelles’ stability and functions anticipate the HSP pathogenesis [152,153]. Further studies are required to clarify the involvement of SWS in the cooperation of the endoplasmic reticulum, mitochondria, LDs and the role of these organelles’ turnover and function in the pathogenesis of HSP and other *NTE/PNPLA6*-associated neurodegenerative syndromes.

## 4. Materials and Methods

### 4.1. Drosophila Stocks and Feeding

For the RNAi-dependent *sws* knockdown, the *y^1^ v^1^; P{TRiP.HMJ23229}attP40* line was used (BDSC №61338, abbreviated in this article as *UAS-sws-RNAi*). This one needs a GAL4 transcription activator for expression because of the *UAS* regulatory sequence presence [34].

*P{GawB}elav^C155^* stock (BDSC №458, abbreviated in this article as *elav-GAL4*) and *y[1], w[1118]; P{y[+t7.7] w[+mC] = nSyb-GAL4.P}attP2* stock (BDSC 51941, abbreviated in this article as *nSyb-GAL4*) were used for induction of expression the GAL4 transcription activator in neurons, whereas *w; +; tub-Gal4/TM3,Sb* (abbreviated in this article as *tub-GAL4*) was used for induction of expression the GAL4 transcription activator in the all fly cells.

*repo-Gal4, UAS-CD8::GFP/TM6B* stock was used for induction of expression the GAL4 transcription activator in glial cells (kindly donated by Mikael Simons, abbreviated in this article as *repo-GAL4*). In addition, to phenocopy the *sws* loss-of-function effects in the nervous system, we generated double driver line *repo, nSyb, UAS-CD8::GFP//TM6B, Sb* for induction of expression the GAL4 transcription activator in both neurons and glial cells.

Additionally, we used the own promotor of *sws* to activate GAL4 expression in cells where *sws* is expressed (KYOTO DGGR 104592, abbreviated in this article as *sws-GAL4*). To visualize cells with an active *sws* promoter, we used *w; UAS-nlsLacZ, UAS-CD8::GFP* stock (kindly donated by Frank Hirth, abbreviated in this article as *UAS-CD8::GFP*).

As controls, *w^1118^, OregonR* and *CantonS* lines were used (St. Petersburg State University fly collection, kindly donated by Elena Golubkova).

To visualize lipid droplets, w*; +; P{w[+mC]UAS-plin2:EGFP}#14A/TM3 Sb[1] e[1]float flies were used (kindly donated by Ronald Kühnlein, abbreviated in this article as *UAS-plin2-EGFP*).

*w[1118]; P{w[+mC] = UAS-mito-roGFP2-Orp1}10* (abbreviated in this article as *UAS-mito-roGFP2-Orp1*) was used for analysis of the hydrogen peroxide relative level in mitochondria. The stock was kindly donated by Jörg Großhans.

*w[1118]; P{w[+mC] = UAS-mito-HA-GFP.AP}2/CyO* (BDSC №8442, abbreviated in this article as *UAS-mito-GFP*) was used for visualization of mitochondria.

In longevity and neurodegeneration level and mushroom body morphology assays, we analyzed *sws^1^* stock Kretzchmar et al., 1997, kindly donated by Doris Kretzschmar.

In all experiments, except the longevity assay, flies were kept at +25 °C on the standard food (35 g semolina, 40 g sucrose, 25 g dry yeast, 4 g agar, 7 mL propionic acid per 1 L of distilled water) for feeding and breeding. In the longevity assay, flies were kept on 2.2% agar with a 100 μL droplet of yeast suspension (2 g of dry yeast diluted in 10 mL of dH_2_O). In the starvation experiment, flies were kept on 1% agar diluted in PBS.

In all experiments, we analyzed only males to avoid sex-specific manifestations that could be different in males and females.

This study was approved by the Ethical Committee of the Petersburg Nuclear Physics Institute, named by B.P. Konstantinov of NRC “Kurchatov Institute” (protocol # 01/KПБ of 13 January 2020).

### 4.2. Quantitative Analysis of mRNA Level

The total mRNA was obtained from 50 male heads using QuickRNA™ Mini Prep (ZymoResearch, Irvine, CA, USA) according to the manufacturer’s protocol. Genomic DNA was depleted using 1 U DNaseI (ThermoScientific, Waltham, MA, USA) application directly to a column matrix. cDNA was synthesized from purified mRNA by reverse transcription using 200 U MMLV-RT (SYNTOL, Moscow, Russia) per the whole total volume of obtained mRNA with both random6 and oligo-dT primers (SYNTOL, Moscow, Russia). Then, qPCR was performed using iTaq Universal SYBR Green Supermix (Bio-Rad, Hercules, CA, USA) in a total volume of 10 μL with 1 μL of obtained cDNA in CFX96 thermocycler (Bio-Rad, Hercules, CA, USA). There were 50 cycles (96 °C—30 s, 57 °C—30 s, 72 °C—30 s) and subsequent melt-curve analysis for verifying the single product presence in each reaction. Primers were common for all known *sws* transcripts (5′ to 3′): ACTACTCAATCATCAAATCTCC and CAGGATTGTGGGTTAATCG. As a reference gene *Gapdh2* was chosen and corresponding primers were the following (5′ to 3′): GATGAGGAGGTCGTTTCTAC and ACCAAGAGATCAGCTTCAC. Measured Cq values from 3 biological and 6 technical replicates were used to assess the relative *sws* transcripts level normalized to the *Gapdh2* mRNA level.

### 4.3. Quantitative Analysis of the NTE-Like Esterase Activity

Two hundred frozen fly heads were homogenized (5% *v*/*v*) in a buffer (50 mM Tris-HCl and 0.2 mM EDTA (pH 8.0)). After centrifugation (9000× *g*; 15 min; +4 °C; Eppendorf Centrifuge 5804R, Hamburg, Germany), the 9S homogenate supernatant was assayed by the differential inhibition colorimetric method [154] with slight modifications [155] as described in detail [156,157]. The NTE-like activity is defined as the portion of phenyl valerate (PV) hydrolyzing activity that is resistant to paraoxon (40 µM, a nonneuropathic organophosphate (OP)) but sensitive to mipafox (1 mM; a neuropathic OP). The concentration of mipafox of 1 mM was chosen here as reaching a plateau when the paraoxon-resistant part of the PV hydrolyzing activity of the 9S supernatant of fly head homogenate was titrated with mipafox. For the assay, the 9S supernatant of fly head homogenates were preincubated (+37 °C; 20 min) with paraoxon in the presence or absence of mipafox before a substrate (PV) was added, and the reaction was allowed to proceed for additional 20 min before stopping by SDS buffer solution with 4-AAP. The endpoint absorbance was measured at 510 nm after K_3_Fe(CN)_6_ adding, using a Bio-Rad Benchmark Plus Microplate Reader (Marnes-la-Coquette, France). The activity was determined as the difference in the amount of phenol released by these reactions and expressed as the NTE-like activity (i.e., the difference in the activity measured in the presence and absence of mipafox) per milligram of protein. Measurements were repeated at least three times for each sample. Protein concentration was assayed with the Coomassie blue dye-binding method and bovine serum albumin as a standard [158]. Four biological replicates were assayed for each genotype.

### 4.4. Preparation and Analysis of Brain Sections

The total neurodegeneration level was assessed in paraffin brain sections. Flies were fixed for 24 h in freshly prepared 4% paraformaldehyde (PanReac AppliChem, Barcelona, Spain). Then, they were transferred through solutions of 70%, 95% and 100% ethanol and methyl ester of benzoic acid (Vecton, St. Petersburg, Russia) and were finally embedded in molten paraffin (Merck, Darmstadt, Germany). The obtained fly brain paraffin sections (6-μm-thick) were stained with hematoxylin (BioVitrum, St. Petersburg, Russia) and eosin (BioVitrum, St. Petersburg, Russia) after deparaffinization. The degree of neurodegeneration was evaluated as the ratio of the total area of vacuoles to the entire area of a brain section, using a Leica DM 2500 (Leica microsystems, Germany) light microscope and the ImageJ software. A total of 82 sample values was obtained from 8–10 flies per genotype and age, with 9–10 good-quality sections per fly.

Semi-plastic brain sections were made as described in our previous work [111].

### 4.5. Mushroom Body Phenotype Analysis

To analyze the frequency of abnormal MB phenotypes, Z-stack confocal images of the entire adult brain were captured using a confocal microscope (Zeiss LSM 700, ZEISS, Oberkochen, Germany). Fly brains were dissected and stained with antibodies to visualize late-born α and β lobes. The numbers of underdeveloped and lost MB lobes were quantified. All experiments were performed at least in two biological replicates for each genotype. For comparison of the observed phenotypes, two-way tables and χ^2^ test were used.

### 4.6. Immunohistochemistry for Mushroom Body Analysis

Fly brains were dissected in PBS and then fixed in 4% formaldehyde diluted in PBS for 15 min at room temperature. Next, brains were washed with PBT (0.2% Triton X-100 in 1× PBS) four times, followed by PBTB administration (2 g/L Bovine Serum Albumin, 5% Normal Goat Serum, 0.5 g/L sodium azide) for 1 h at room temperature, and then incubated at +4 °C in with primary antibodies diluted in PBTB on nutator overnight. The following day, samples were washed with PBT four times, followed by block for 1 h and 2 h incubation with secondary antibodies at room temperature. Next, samples were washed four times with PBT (one of the washes contained DAPI to stain nuclei). Lastly, a medium (70% glycerol, 3% n-propyl gallate in 1× PBS) was added to samples for later mounting on the slides. The following primary antibodies were used: mouse anti-FasII (1:25) from Developmental Studies Hybridoma Bank (DSHB, Iowa City, IA, USA), chicken anti-GFP (#ab13970, 1:1000, Abcam, Cambridge, UK). The following secondary antibodies were used: goat anti-chicken Alexa 488 (1:500, A-11039, Life Technologies, Thermo Fisher Scientific, Waltham, MA, USA) and goat anti-mouse Cy3 IgG1 (1:250, Jackson ImmunoResearch Laboratory, West Grove, PA, USA). For visualization of cell nuclei, DAPI dye was used (1:1000, Sigma, Merck, Darmstadt, Germany). Samples were analyzed using a confocal microscope (Zeiss LSM 700, ZEISS, Oberkochen, Germany). For making figures, Adobe Photoshop software was used.

### 4.7. Survival Analysis

One- to two-day-old imagoes were placed in test tubes with agar and yeast suspension and were kept at +25 °C (30–40 males per a vial, with a total of at least 300 males per genotype). Live flies were flipped to a new vial with a fresh medium every 2–3 days and the number of dead individuals was counted. The experiment was conducted until the death of the last fly.

### 4.8. Survival Analysis of Starved Flies

Zero- to twelve-hour-old imagoes were collected at 10 a.m./p.m. and placed in test tubes with agar and were kept at +25 °C (30–40 males per a vial, with a total of at least 150 males per a genotype). Every 12 h (at 10 a.m./p.m.), the number of dead flies was counted without flipping the flies to a new vial. The experiment was conducted until the death of the last fly.

### 4.9. Negative Geotaxis Assay

Locomotor activity and negative gravity taxis were tested using the RING assay, as described in [159]. Briefly, six groups of 20–40 flies of each genotype and age were transferred into empty 50 mL falcons without anesthesia, and the vials were loaded into the RING apparatus. The apparatus was rapped three times in rapid succession to initiate a negative geotaxis response. The flies’ movements in tubes were videotaped and digital images captured 3 s after initiating the behavior. The distance between a fly and a vial bottom was calculated for each fly. The performance of flies was analyzed in six consecutive trials (interspersed with a 60 s rest). For each genotype and age, more than 200 flies participated in the experiment, and for each fly, six replicate values were obtained. To equalize the total sample size, 2000 values were chosen randomly among the obtained data sample for every genotype and age.

### 4.10. Memory Index Assessment

Around 20–30 males per vial were kept until the standard protocol for the olfactory learning and memory test was applied [160,161]. Briefly, the experiment was performed in a temperature-controlled dark room using T-maze, with one odorant (3-octanol or 4-methylcyclohexanol (Fluka, Sigma-Aldrich, Buchs, Switzerland)) having been compiled with 12 electric stimuli (60 V, 80 mA) for 1 min during training, while the other odorant had not. After a pause of 60 min, flies were allowed to go into two tubes of T-maze, each containing one of the odorants, for 2 min. Finally, the number of flies that stayed in each tube was counted and the memory half-index was calculated for each vial, as described in [160]. For both tests with octanol and 4-methylcyclohexanol, nine biological replicates were analyzed (i.e., nine vials with 20–30 flies for each of the odorants). Then, we generated 81 independent pairs of the memory half-indices, resulting in a sample size of 81 means (memory indices per se). For each age and genotype, more than 600 flies participated in the experiment.

### 4.11. Transcriptomic Assay

Total RNA was extracted from twenty 30-day-old males with the RNeasy Mini Kit (QIAGEN, Hilden, Germany) with two replicates per genotype, and equal amounts of purified good-quality RNA were then prepared for hybridization using the 3′IVT PLUS Express kit (900720, Affymetrix, Thermo Fisher Scientific, Waltham, MA, USA), yielding a fragmented biotin-labeled cRNA library. After adding internal probe array controls to the target samples for signal normalization, the mixtures were hybridized with the GeneChip *Drosophila* 2.0 (Affymetrix, Thermo Fisher Scientific, Waltham, MA, USA), and then washed and stained with the Hybridization, Wash and Stain Kit (900720, Affymetrix, Thermo Fisher Scientific, Waltham, MA, USA). All procedures were performed according to the manufacturer’s protocol. After scanning fluorescent signals from cRNA-chip matrix heteroduplexes, data transformation was conducted with the RMA algorithm and quality control was performed using GeneChip Expression Console Software 1.4 (Affymetrix, Thermo Fisher Scientific, Waltham, MA, USA). Eventually, lists of up- and downregulated genes in knockdown flies compared to control flies were obtained, assessing more than 18,500 transcripts with TAC4.0 (Affymetrix, Thermo Fisher Scientific, Waltham, MA, USA). Functional enrichment analysis of these data files was performed with g:Profiler [162]. For analysis, only the genes with more than a two-fold change in expression (False Discovery Rate (FDR) < 0.05) were considered and the gene groups divided by the Gene Ontology (GO) biological process were studied (adjusted *p*-value < 0.05).

### 4.12. Oxidative Particle Measurement

The reactive oxygen species (ROS) level was measured using 2′,7′-dichlorodihydrofluorescein diacetate (H2DCF-DA, Invitrogen, Thermo Fisher Scientific, Waltham, MA, USA), as described in [163]. Twenty heads for each biological replicate, among seven total replicates, were homogenized in a buffer containing 100 μL of 10 mM Tris (pH 7.4) and 3 μL protease inhibitor (Roche, Basel, Switzerland). The homogenate was centrifuged (for 10 min at 10,000× *g* rpm, +4 °C). Then, 5 μL of clear supernatant was mixed with 60 μL of 5 μM H2DCF-DA and incubated for 60 min at +37 °C, and the procedure was repeated three times to obtain three technical replicates for each biological replicate. The fluorescence emission of DCF resulting from H2DCF-DA oxidation and hydrolysis was scanned at 485 nm excitation and 530 nm emission with a plate reader (EnSpire2300, PerkinElmer, Waltham, MA, USA). The values obtained for ROS levels in three technical replicates were normalized to the protein concentration measured by the standard protocol of the Bradford assay and then were averaged.

### 4.13. Hydrogene Peroxide Relative Level Assessment

To assess the relative level of hydrogen peroxide in the brain neuronal mitochondria, the special sensor transgenic stock was used (*UAS-mito-roGFP2-Orp1*), as described in [164,165]. Briefly, 12 fly brains were incubated in 20 μM N-ethyl maleimide (Sigma-Aldrich, Waltham, MA, USA) for 10 min. Then, they were washed in phosphate-buffered saline (PBS, BioVitrum, St. Petersburg, Russia), fixed in 4% formaldehyde solution for 10 min, washed in PBS and finally placed in Antifade Mounting Medium (Vectashield, Vector Laboratories, Burlingame, CA, USA). The same numbers of brains were preliminarily incubated for 10 min with 100 μM diamide (Sigma-Aldrich, Waltham, MA, USA) to oxidize the sensor or with 10 μM dithiothreitol (Sigma-Aldrich, Waltham, MA, USA) to reduce the sensor, and brains were then washed in PBS. All brain samples were analyzed with the Leica LX laser confocal microscope using a 20× (oil) objective at 405 or 488 nm excitation and 500–530 nm emission, resulting in a series of images (thickness of 100 μm, and distance between images of 2 μm). A quantitative analysis of the total fluorescence in 3D reconstructed images was performed with the ImageJ software (function: “Total Grey Area”). The sample values for analysis were calculated as the normalized 405/488 ratios of the average fluorescence levels.

### 4.14. Brain Mitochondria Analysis

Heads of adult flies were separated from bodies and brains were isolated and fixed in a freshly prepared 4% paraformaldehyde for 20 min, followed by washing in PBS 3 × 5 min. The brains were placed in the Antifade Mounting Medium (Vectashield, Vector Laboratories, Burlingame, CA, USA). Series images (2-μm-thick) were obtained with the Leica LX laser confocal microscope (Leica microsystems, Wetzlar, Germany) using 40× oil immersion objective and 488 nm laser excitation. The mito-GFP fluorescence intensity in an adult brain was defined as the mean grey value on an area of the cell bodies, dendrites, axons of a mushroom body or antennal lobes using the ImageJ software. To determine the mitochondrial circularity index, a 63× oil objective and the same excitation wavelength were used. Z-stack images were captured at a slice interval of 0.10 μm in the calyx and bulbous tip of the α lobe central location from an anterior view (Figure 5, indicated by white boxes). The mitochondrial circularity index was determined with the ImageJ software using the Shape Descriptors plugin and the formula for quantifying:

$$Circularity=4\pi \frac{Area}{Perimeter^{2}}$$

(Values must be within the range from 0 to 1, where 1 corresponds to a circle; as the shape becomes more elongated, the value approaches zero).

### 4.15. ATP Relative Level Determination

The ATP level was evaluated with the ATP determination kit (A22066, Molecular Probes, Invitrogen, Thermo Fisher Scientific, Waltham, MA, USA) according to [166], with modifications. Males were incubated at +25 °C till the 5th or the 25th day, frozen in liquid nitrogen, and 30 heads were collected at +10 °C, frozen in liquid nitrogen again and homogenized in a lysis buffer (6 M guanidine HCl, 100 mM Tris (pH 7.8), 4 mM disodium EDTA). Then, the sample was boiled for 5 min, centrifuged (+10 °C, 14.1× *g* rcf, 3 min), and 10 μL was dissolved in 90 μL of a dilution buffer (25 mM Tris (pH 7.8), 100 μM disodium EDTA) and centrifuged again (+10 °C, 14.1× *g* rcf, 3 min). Next, 10 μL of the sample was added to a well of the plate with 100 μL of the ATP determination reagent. The total luminescence was measured using a plate reader (EnSpire2300, Perkin Elmer, Waltham, MA, USA) 5 times for each replicate. The experiment was conducted in 5 biological replicates and 4 technical replicates, so that the total size was 100 values in a sample.

### 4.16. Mitochondria Analysis in Wing Axons

For analysis of the wing nerve mitochondria, wings were removed from anesthetized flies, using Vannas Spring Scissors (Cutting Edge 3mm, # 15000-10, Fine Science Tools, Heidelberg, Germany), and placed in a drop of Halocarbon Oil 700 (CAS 9002-83-9, Sigma, St. Louis, MO, USA) on a glass slide. The sample was covered with a coverslip and immediately analyzed with the Leica LX laser confocal microscope (Leica microsystems, Wetzlar, Germany) using 63× (oil) objective and 488 nm laser excitation, having obtained 1-μm-thick image series. The total number of mitochondria in the wing neurons was calculated using the Shape Descriptors plugin of the ImageJ software. The circularity indices were determined as described above (see Section 4.14). The mobile mitochondria numbers were analyzed using the Cell Counter plugin (https://imagej.net/Cell_Counter (accessed on 31 July 2021)).

### 4.17. Axonal Transport Assay

Axonal transport analysis was performed for the wing neurons of the adult flies (section “a”, vein L3, Figure S6B). Samples were prepared according to the protocol [167]. Live video recording was performed with a Leica LX laser confocal microscope (Leica microsystems, Wetzlar, Germany) using 63× (oil) objective. The optimal parameters were chosen for recording (image size—1024 × 150, frequency 400 Hz, scanning format—XYT, shooting time—2 min at the speed of 2 frames/s). The average mitochondrial velocity was analyzed using the MTrackJ plugin (www.imagescience.org/meijering/software/mtrackj, accessed on 27 July 2021), which allows tracking of the movement of organelles manually (Figure S6C).

### 4.18. Lipid Droplet Visualization and Quantitative Analysis

For visualization of lipid droplets, we used the *UAS-plin2-EGFP* transgene [58]. At least 30 brains for every genotype were dissected in cold PBS, fixed in 4% solution of paraformaldehyde for 15 min and rinsed in PBS for 10 min three times. Then, the brain samples were placed in Antifade Mounting Medium (Vectashield, Vector Laboratories, Burlingame, CA, USA) for same-day imaging. Series of images (total thickness 60 μm, distance between images 2 μm) were obtained with the Leica LX confocal microscope (Leica microsystems, Wetzlar, Germany). For lipid droplet number analysis, central brain regions in 3D reconstructed confocal images of the 387.50 × 387.50 µm area were analyzed, using the ImageJ software (“Analyse Particles” function) to quantify particles with the size ≥0.4 pixel2. This method allowed us to avoid a tissue autofluorescence-induced bias. For lipid droplet size analysis, single confocal images for glial LDs and 3D stacks for neuronal LDs at similar coordinates in the central brain on an area of 273 × 273 µm (63× objective) were chosen. The minimal size of 0.4 μm^2^ was selected for identifying single lipid droplets. For each brain, the total number of lipid droplets and the size of each of them were determined. Then, we divided the lipid droplet size into 4 groups, 0.4–1 μm^2^, 1–2 μm^2^, 2–3 μm^2^, >3 μm^2^, and calculated the proportion of lipid droplet numbers of a certain size in each brain.

### 4.19. Results Processing

Statistical analysis was performed using KyPlot 5.0 software. All samples were tested for normality with the Shapiro–Wilk test. If the distribution was normal, we used parametric tests: the Student *t*-test for a comparison of two samples and Dunnett’s test for multiple comparisons (three samples). For normally distributed samples, data were presented as histograms (mean ± 95% confidence interval (CI)). For other distribution types, nonparametric tests were used. For a comparison of two samples, the Mann–Whitney test was applied, whereas for multiple comparisons (three samples), the Steel test was performed. For samples where the Shapiro–Wilk test suggested a non-normal distribution, data were presented as box-and-whisker plots. In most cases where there were several technical replicates, we did not average them, but put all the values in one sample, assuming that each value is an independent representative of an entire assembly of a studied parameter (an exception was ROS analysis, described in Section 4.9).

## Data Availability

The data presented in this study are available in the article and Supplementary Materials.

## Supplementary Materials

The following are available online at https://www.mdpi.com/article/10.3390/ijms22158275/s1. Figure S1: Analysis of the *UAS-sws-RNAi^v5469^* (VDRC v5469) transgene effects, Figure S2: Downregulation of *sws* in glia (*repo-GAL4;UAS-sws-RNAi*) results in the mushroom body defects. Figure S3: GO BP processes under the control of downregulated genes in 25-day-old neuronal *sws* knockdown males (*elav-GAL4;UAS-sws-RNAi*) compared to *CantonS* control and respective FDR-adjusted *p*-values of functional enrichment analysis in g:Profiler software, Figure S4: GO BP processes under the control of downregulated genes in 25-day-old neuronal *sws* knockdown males (*elav-GAL4;UAS-sws-RNAi*) compared to *CantonS* control and respective query gene number from functional enrichment analysis in g:Profiler software, Figure S5: GO BP processes under the control of upregulated genes in 25-day-old neuronal *sws* knockdown males (*elav-GAL4;UAS-sws-RNAi*) compared to *CantonS* control and respective FDR-adjusted *p*-values of functional enrichment analysis in g:Profiler software, Figure S6: GO BP processes under the control of upregulated genes in 25-day-old neuronal *sws* knockdown males (*elav-GAL4;UAS-sws-RNAi*) compared to *CantonS* control and respective query gene number from functional enrichment analysis in g:Profiler software, Figure S7: Analysis of axonal transport in the wing neuron, Figure S8: Analysis of lipid droplets in glia of the fly brain. Data S2: Proteome analysis method and results description. Data S3: Lists of proteins found to be up/downregulated in heads of *sws^1^* mutants identified by the proteomic analysis and genes found to be up/downregulated in organism of *elav-GAL4; UAS-sws-RNAi* flies identified by the transcriptomic analysis.
