# Supplementary material for: Loss of swiss cheese in Neurons Contributes to Neurodegeneration with Mitochondria Abnormalities, Reactive Oxygen Species Acceleration and Accumulation of Lipid Droplets in Drosophila Brain
Source: Int J Mol Sci. 2021 Jul 31;22(15):8275. doi: 10.3390/ijms22158275 (PMC8347196; doi:10.3390/ijms22158275)
Supplement: Supplementary file 1 [file ijms-22-08275-s001.zip › Supplementary Data 2.pdf]

Supplementary Material 2

Proteome Analysis Method

For proteomic assay, we collected 50 male heads of mutant (*sws*[1]) or control (*CantonS*) genotypes, the age of flies was 25 days. We prepared 3 samples per each genotype (3 biological replicates). The heads were rubbed with a pestle on liquid nitrogen, then the Rabilloud buffer (7 M urea, 2 M thiourea, 4% CHAPS, 1% DTT, cocktail of protease inhibitors) was added. The total amount of protein in an aliquot was measured by the Bradford method.

The lysate (30 µL) was transferred to concentration filters (Microcon devices YM-30, Merck, USA), then 100 µL of reducing solution (100 mM DTT, 100 mM Tris-HCl, pH 8.5) was added and the mix was incubated for 1 hour at 56 ˚C with periodic shaking and centrifuged (hereinafter: shaking for 30 s – 600 rpm, 300 s – no shaking, Thermomixer Comfort (Eppendorf, Germany); centrifugation at 9000 rcf for 15 min at room temperature, MiniSpin Centrifuge (Eppendorf, Germany)). The filters were washed 3 times (200 µL of wash buffer (8 M urea, 100 mM Tris-HCl, pH 8.5) was applied and centrifugation was performed at 9000 rcf for 15 min at the room temperature).

Then alkylating buffer (50 mM iodoacetamide in wash buffer) was applied to the filter. It was incubated for 1 hour at 25 ˚C with periodic shaking and centrifuged. The filters were washed 3 times again.

Then the filters were washed 2 times by adding 200 µl of trypsinolysis buffer (50 mM ammonium bicarbonate, pH 8.5), centrifuged at 9000 rcf for 15 min at the room temperature. After that, 100 µl of trypsinolysis buffer was applied to the filter and trypsin solution (Sigma Aldrich, Merck, USA) was added (the amount of enzyme was 100 times less than the total amount of protein). The mix was incubated overnight at 37 ˚C with periodic shaking.

Then the samples were centrifuged at 9000 rcf for 15 min using Varifuge 3.0RS centrifuge (Heraeus Instrument, Germany) at a temperature of 20 ˚C. After that 50 µL of formic acid solution (30%) was added and filters were centrifuged at 9000 rcf for 15 min using the Varifuge 3.0RS centrifuge at a temperature of 20 ˚C. The resulting solution of peptides was dried using SpeedVac Vacuum Concentrator (ThermoFisher Scientific, USA) and then the sample was dissolved in 5% formic acid to obtain a concentration of 1 µg/µL (it was assumed that the total mass of peptides is equal to the total mass of the total protein concentration).

Using an Agilent HPLC system 1100 Series (Agilent Technologies, USA), 4 µg of peptides in 5% (v/v) formic acid were injected onto a trap column Zorbax 300SB–C18, 5x 0.3mm (Agilent Technologies, USA). After washing with 5% (v/v) acetonitrile 0.1% (v/v) formic acid peptides were resolved on a 150 mm x 75 µm Zorbax 300SB-C18 reverse phase analytical column (Agilent Technologies, USA) over a 30 min organic gradient of 5-60% CH_3CN, 0.1% formic acid with a flow rate of 300 nl/min. Peptides were ionized by nano-electrospray ionization at 2.0 kV using a fused silica emitter with an internal diameter of 8 µm (New Objective, USA). Tandem mass spectrometry analysis was carried out on an Orbitrap Velos mass spectrometer (ThermoFisher Scientific). Mass spectra were acquired in positive ion mode. High resolution data was acquired in an Orbitrap analyzer with a resolution of 30,000 (m/z 400) for MS and 7,500 (m/z 400) for MS/MS scans. Survey MS scan was followed by MS/MS spectra of the five most abundant precursors. For peptide fragmentation higher energy collisional dissociation (HCD) was set to 35 eV, the signal threshold was set to 5000 for an isolation window of 2 m/z and the first mass of HCD spectra was set to 100 m/z. Fragmented precursors were dynamically excluded from targeting for 90 seconds. Singly charged ions and ion with not defined charge state were excluded from triggering MS/MS scans. The automatic gain control target value was regulated at 1 × 106 with a maximum injection time of 100 ms and at 1 × 105 with a maximum injection time of 250 ms for MS and MS/MS scans, respectively.

Data were searched, using the Mascot search engine (www.matrixscience.com) with the following parameters: enzyme = trypsin (allowing for cleavage before proline); maximum missed cleavages = 2; fixed modifications = carbamidomethylation of cysteine; variable modifications = oxidation of methionine; phosphorylation of serine and threonine, acetylation of lysine; precursor mass tolerance = 10 ppm; product mass tolerance = 0.1 Da. NCBInr database was used as a protein sequence database. For FDR assessment, a separate decoy database was generated from the protein sequence database. False positive rate of 0% was allowed for protein identification.

The quantitative analysis of the protein representation was carried out according to the obtained emPAI values, normalized to the total protein concentration in the sample and the total emPAI. The amount for different proteoforms and isoforms of a one protein was averaged. Each biological replication was analyzed in three technical replicates, the obtained quantitative data were averaged.

For functional annotation g:Profiler (https://biit.cs.ut.ee/gprofiler/gost), STRING 11.0 (https://string-db.org/) and DAVID 6.8 (https://david.ncifcrf.gov/) online resources were used.

Results Summary

Using the method of a tandem mass spectrometry of chromatographically separated peptides obtained after trypsinolysis of fly head lysates, 1968 proteins were detected in 25-day-old *sws¹* males and 1724 proteins in the same aged *CantonS* males. Of these proteins, 1445 were found in both variants. In groups of differentially represented proteins we took those ones, the level of which in the mutant was 2 or more times higher (or less) than in the wild-type flies.

To the group of proteins, the level of which in the mutant was 2 or more times higher than in the wild-type males (62 proteins), we also added proteins which were detected only in the mutant, but not in the control (523 proteins). We did this because of an assumption that control individuals may have such proteins, but the amount of them is less than the threshold level of protein detection in our method. Thus, 585 proteins with an increased content in the heads were found in the mutant compared to the control.

To the group of proteins, the level of which in the mutant was 2 or more times less than in the wild-type males (48 proteins), we also added proteins which were detected only in the control, but not in the mutant (279 proteins). We did this because of assumption that mutants may have such proteins, but the amount of them is less than the threshold level of protein detection in our method. Accordingly, it was considered that the amount of 327 proteins in the heads of the mutant flies was reduced in comparison with the control.

Full lists of these two groups of proteins are accessible in the Supplementary Material 3.

Functional Analysis of the Proteins, the Level of which is Increased in the Mutant Compared to the Control

Of the 585 proteins in this group, 542 proteins were annotated in the GO BP database (according g:Profiler analysis). The detected proteins have different molecular functions (GO MF), possessing oxidoreductase, isomerase, transferase, transaminase, dehydrogenase, lyase, hydrolase activities (p_{adj} < 0.05). Proteins are capable of binding ions, nucleotides, nucleotide phosphates, carbohydrates, estradiol and testosterone, ribonucleotides, steroids, vitamins, NAD(P)H. Localization of the detected proteins in a cell is diverse: cytosol, mitochondria, endoplasmic reticulum, nuclear membrane, vesicle pubescence, axons, blood-brain barrier (GO CC).

The detected proteins are involved in various biological processes (GO BP, 1e-36 < p_{adj} < 0.05): metabolism of small molecules, carboxylic acids, amino acids, carbohydrates, hexoses, nucleic bases and purine-containing compounds, ribonucleosides. Some of the detected proteins (their number is indicated in parentheses) are involved in the assembly and structural organization of intercellular contacts: apical, septate, dense (14); in clathrin-mediated endocytosis (8); visual function (12); oxidative phosphorylation (16); folding of proteins (18); biosynthesis of glutamine and its derivatives (6); metabolism of glycine (8), pyruvate (12), IMP (6), ATP (26).

Analysis of the group of proteins using the STRING resource allowed us to identify highly significant interactions, annotated in databases, of any pair of proteins, as well as to group proteins based on the identified interactions. As a result of the analysis, groups of interacting proteins were identified. In these clusters there were proteins that are responsible for the following functions: vesicular transport, differentiation of muscle and nerve cells, neuron-specific regulation of translation, energy metabolism, metabolism of amino acids and their derivatives, metabolism of phospholipids, protein synthesis, cellular signaling (Figure SS1).

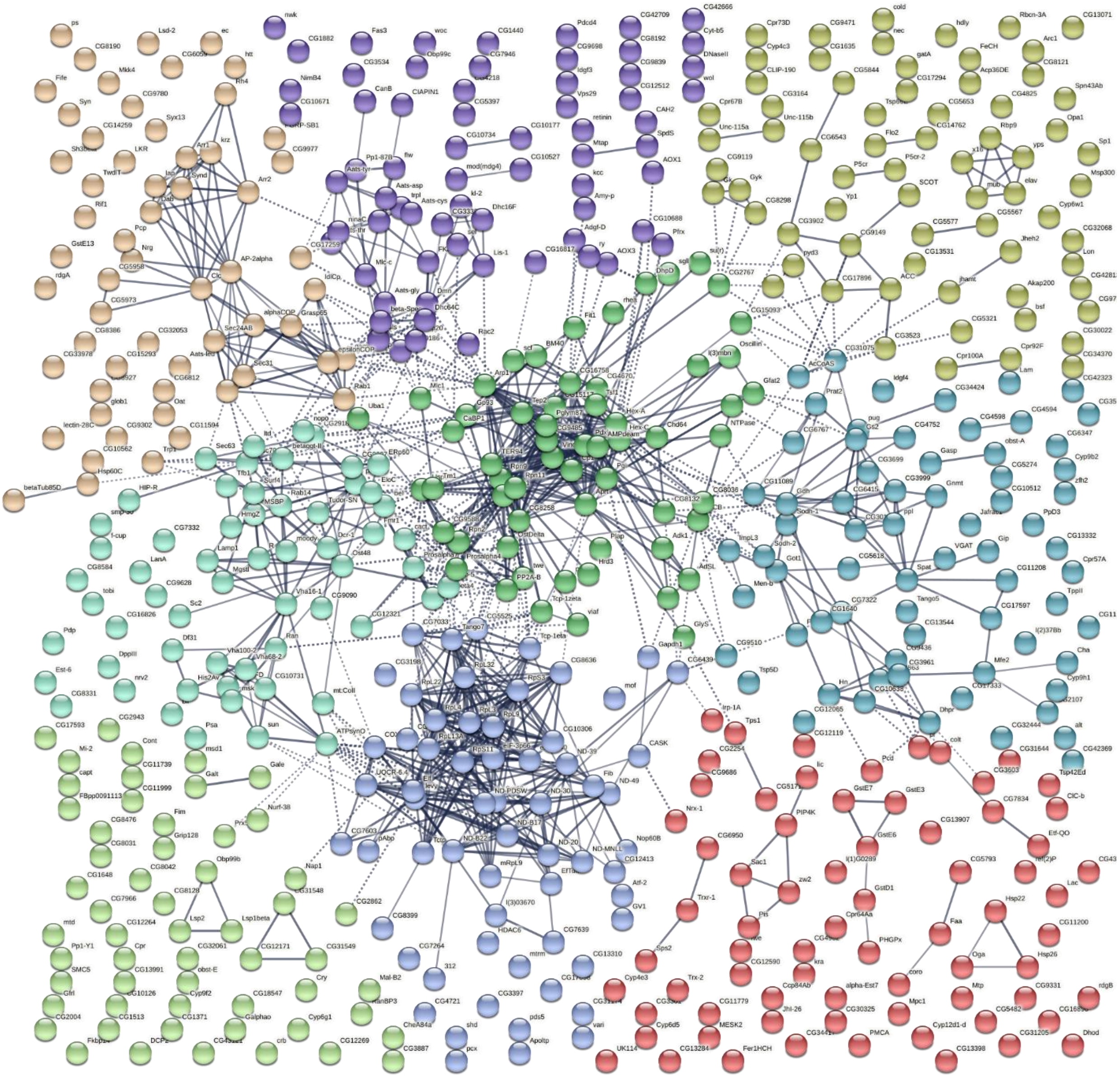

Figure SS1. 585 proteins, the level of which is increased in the mutant, and their physical and functional interactions. The settings for visualization were the following: Active interaction sources – Experiments, Databases, Co-expression; Minimum required interaction score: Highest confidence (0.900); Kmeans clustering = 10.

66 proteins of the group corresponded to the genes which expression was increased in the organism of individuals with *sws* knockdown in neurons (according to the results of transcriptome analysis). Functional activity of these proteins is demonstrated in the Table SS1.

Table SS1. Upregulated proteins revealed by both proteomic and transcriptomic analysis.

**Protein name**	**Function (according to DAVID functional annotation tool)**
ACCCOAS	Acetyl-CoA ligase
ARR1	Arrestin
CG10562	CHK kinase
CG10863	Aldo/keto reductase
CG12171	Glucose/ribitol dehydrogenase
CG12321	Proteasome assembly chaperone 2
CG12512	Long-chain fatty acid-CoA ligase
CG15093	Phosphogluconate dehydrogenase
CG15293	Unknown
CG1648	Lipid particle protein
CG16898	CHK kinase
CG17294	Nitrophenylphosphatase
CG17333	6-Phosphogluconolactonase
CG17597	Acetyl-CoA C-acyltransferase / Thiolase
CG18547	Aldo/keto reductase
CG2107	Carnitine O-palmitoyltransferase
CG31205	Serine-type endopeptidase
CG31548	Glucose/ribitol dehydrogenase
CG3301	Glucose/ribitol dehydrogenase
CG3397	Aldo/keto reductase
CG34424	5-Formyltetrahydrofolate cyclo-ligase
CG3603	Glucose/ribitol dehydrogenase
CG4598	Dodecenoyl-CoA delta-isomerase
CG4721	Metalloendopeptidase
CG5321	Gamma-butyrobetaine dioxygenase
CG6415	Aminomethyltransferase
CG6543	Enoyl-CoA hydratase
CG7322	Glucose/ribitol dehydrogenase

CG7966	Selenium-binding protein, six-bladed beta-propeller
CG8132	Nitrilase
CG9510	Aargininosuccinate / Fumarate lyase
PGRP-SB1	N-acetylmuramoyl-L-alanine amidase
CRYS	Structural constituent of eye lens
CPR57A	Structural constituent of chitin-based larval cuticle
CYP4E3	Cytochrome P450 monooxygenase
CYP9B2	Cytochrome P450 monooxygenase
CYP9H1	Cytochrome P450 monooxygenase
DNASEII	DNase II
DHPD	Dihydropterin deaminase
ETF-QO	Electron-transferring-flavoprotein dehydrogenase
GIP	Xylose isomerase
GSTD1	Glutathione transferase / peroxidase
GSTE7	Glutathione transferase / peroxidase
GNMT	Glycine/Sarcosine N-methyltransferase
HSP26	Myosin binding alpha crystallin / Heat shock protein
HN	Phenylalanine 4-monooxygenase
HEX-C	Hexokinase
LSP2	Larval serum protein complex
MGSTL	Microsomal glutathione S-transferase
MTP	Phosphatidylcholine transporter
OBP99B	Odorant-binding protein
OBP99C	Odorant-binding protein
PRAT2	Amidophosphoribosyltransferase
P5CR-2	Pyrroline-5-carboxylate reductase
RH4	Rhodopsin
SPARC	Calcium ion and collagen binding osteonectin-like extracellular matrix protein
SMP-30	Gluconolactonase
SODH-2	Sorbitol dehydrogenase
TEP2	Alpha-2-macroglobulin
TSF1	Transferrin
GLOB1	Globin

INAD	Receptor signaling complex scaffold rhabdomere protein
NEC	Serpin
PUG	Formate-tetrahydrofolate ligase
TRPL	Transient receptor potential channel
UK114	Enamine/imine deaminase, YjgF-like, endoribonuclease

Functional Analysis of the Proteins, the Level of which is Decreased in the Mutant Compared to the Control

Of the 327 proteins we isolated into this group, 300 proteins were annotated in the GO BP database (according to the g:Profiler resource). The proteins have different molecular functions (GO MF), exhibiting oxidoreductase activity (hereinafter, $p_{adj} < 0.05$). Proteins can bind adenyl nucleotides, nucleoside phosphates, ribonucleotides, carbohydrate derivatives, tropomyosin, ions, also they can participate in structural organization of the ribosome. Localization of the detected proteins in a cell is diverse: cytosol, ribosome, presynaptic active zones, cell contacts, myofibrils, cell body, basement membrane (GO CC).

The detected proteins are involved in various biological processes (GO BP, $1e\text{-}5 < p_{adj} < 0.05$): translation (18), regulation of transport (24), energy metabolism (20), differentiation of myocytes (9).

Analysis of the identified proteins using the STRING resource reveals several interaction groups in which proteins are responsible for the following functions: translation and organization of the ribosome, ubiquitinylation and proteasome degradation, organization of the extracellular matrix, mitochondrial respiratory chain activity, nerve impulse conduction, mRNA splicing and translation regulation (Figure SS2).

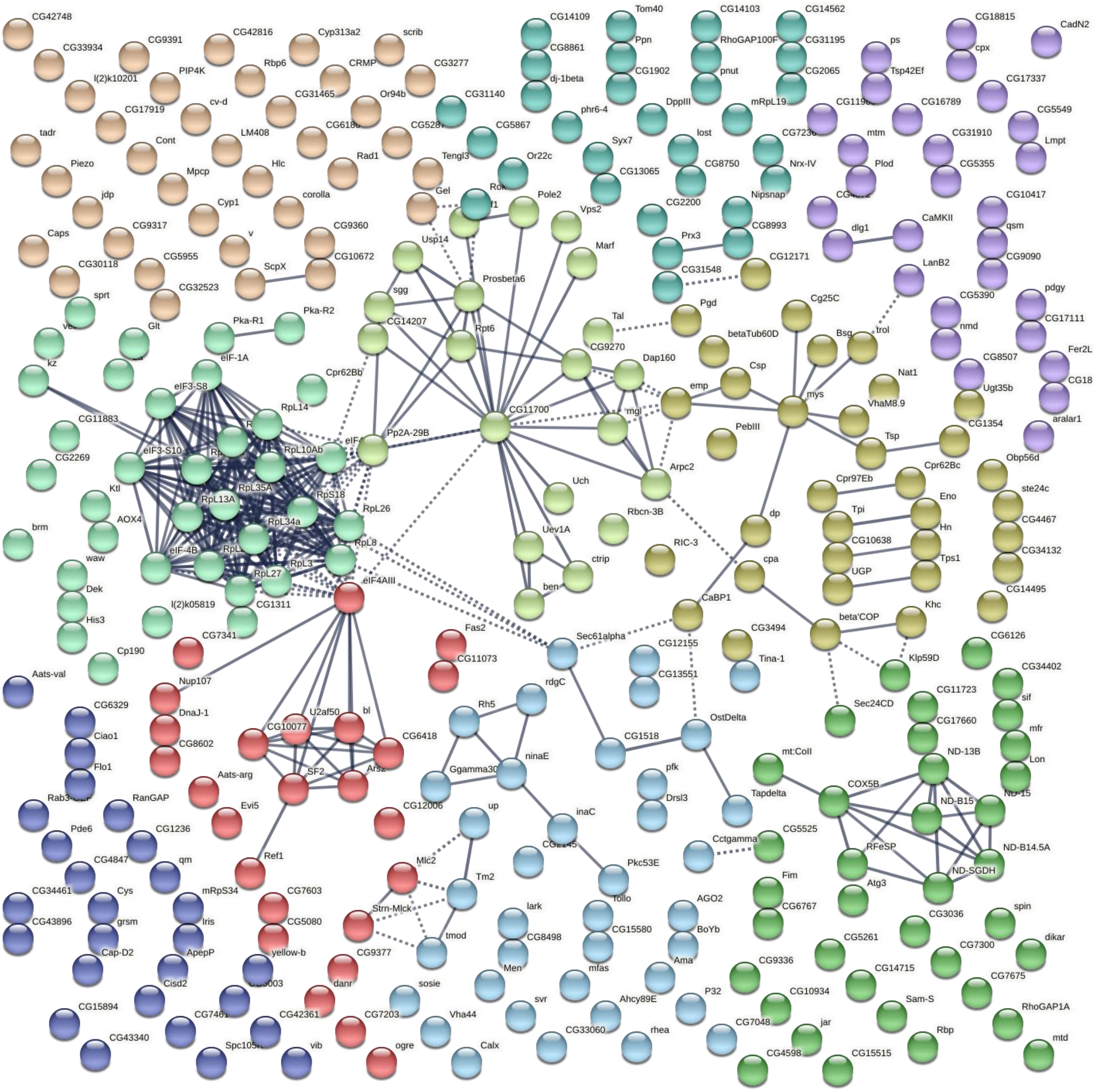

Figure SS2. 327 proteins, the level of which is decreased in the mutant, and their physical and functional interactions. The settings for visualization were the following: Active interaction sources – Experiments, Databases, Co-expression; Minimum required interaction score: Highest confidence (0.900); Kmeans clustering = 10.

12 proteins of the group corresponded to the genes which expression was decreased in the organism of individuals with *sws* knockdown in neurons (according to the results of transcriptome analysis). Functional activity of these proteins is demonstrated in the Table SS2.

Table SS2. Downregulated proteins revealed by both proteomic and transcriptomic analysis.

**Protein name**	**Function (according to DAVID functional annotation tool)**
CG10077	RNA helicase
CG10934	Unknown
CG15580	Unknown
CG31465	Unknown
CG33934	Sodium/sulphate symporter
DIKAR	Bromodomain-containing protein
DRSL3	Knottin
DAP160	EF-hand domain containing protein
SPC105R	Condensed chromosome outer kinetochore
TENGL3	DNA/RNA non-specific endonuclease
TSP	Thrombospondin
STE24C	Metalloendopeptidase
