# Supplementary material for: Loss of swiss cheese in Neurons Contributes to Neurodegeneration with Mitochondria Abnormalities, Reactive Oxygen Species Acceleration and Accumulation of Lipid Droplets in Drosophila Brain
Source: Int J Mol Sci. 2021 Jul 31;22(15):8275. doi: 10.3390/ijms22158275 (PMC8347196; doi:10.3390/ijms22158275)
Supplement: Supplementary file 1 [file ijms-22-08275-s001.zip › Supplementary Figures.pdf]

Supplementary material 1

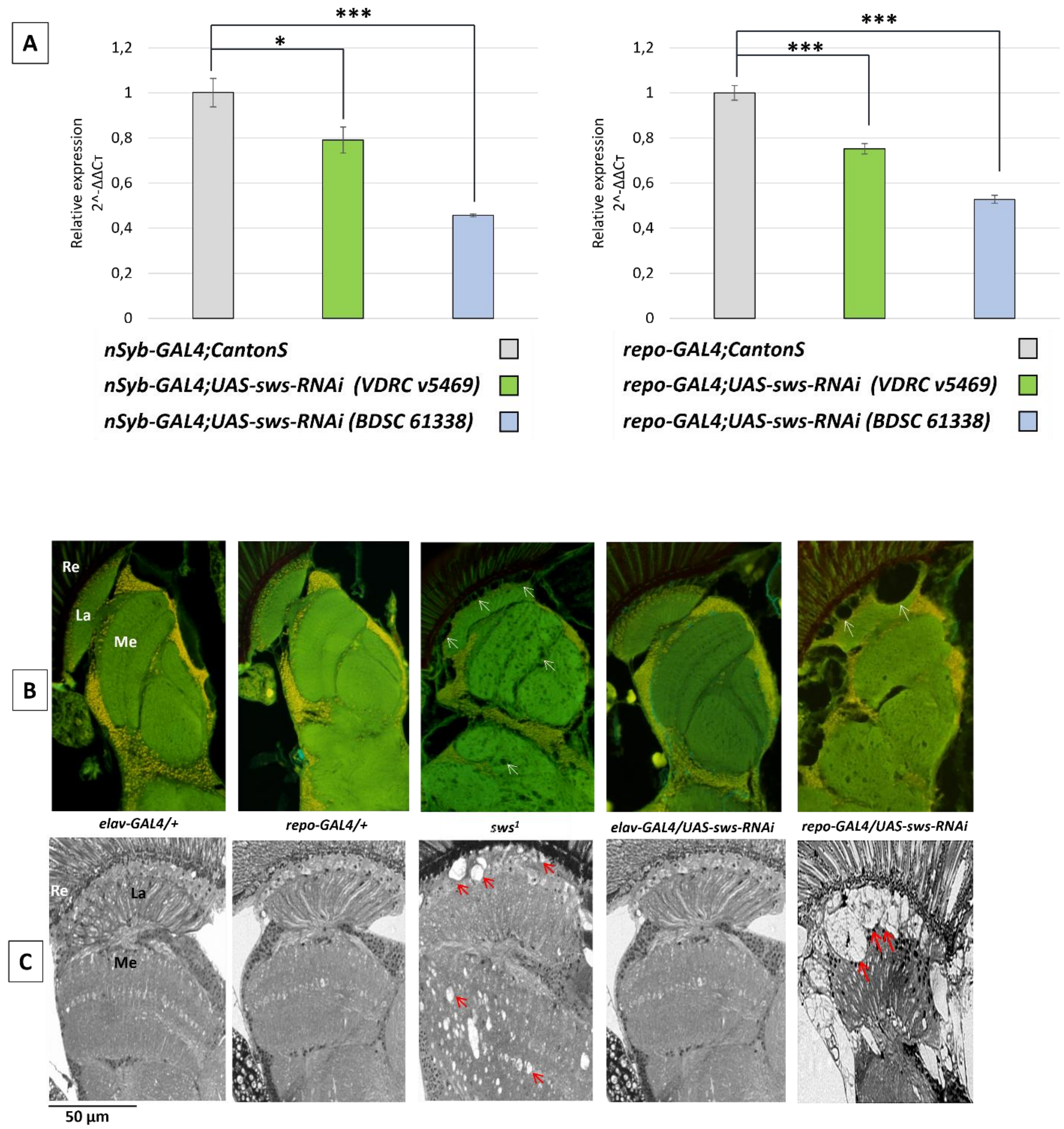

Figure S1. Analysis of the *UAS-sws-RNAi*^{v5469} (VDRC № v5469) transgene effects. (**A**) Relative *sws* mRNA level in heads of flies with *sws* knockdown in neurons (*nSyb-GAL4;UAS-sws-RNAi*) or in glia (*repo-GAL4;UAS-sws-RNAi*) and control flies (F1 males obtained from crossing a *CantonS* female and a respective *GAL4* male). The level of *sws* mRNA in the control is taken as 1,00 in each sample. The mean value of $2^{-\Delta\Delta CT}$ and 95% CI, Student t-test, * $p < 0.05$, *** $p < 0.001$, $N = 3$. (**B**) Horizontal 7 μm paraffin and (C) horizontal 1 μm plastic semithin brain sections of 20-day-old *sws*^{1} mutants, flies with the neuronal (*elav-GAL4;UAS-sws-RNAi*^{v5469}) and glial (*repo-GAL4;UAS-sws-RNAi*^{v5469}) *sws* knockdown or without it (*elav-GAL4*/+ and *repo-GAL4*/+). Re – retina, La – lamina, Me – medulla. Scale bar: 50 μm. Brain tissue neurodegenerative vacuoles are marked with arrows. Flies of the *elav-GAL4;UAS-sws-RNA*^{v5469} showed no neurodegenerative phenotype in contrast to *repo-GAL4;UAS-sws-RNA*^{v5469}.

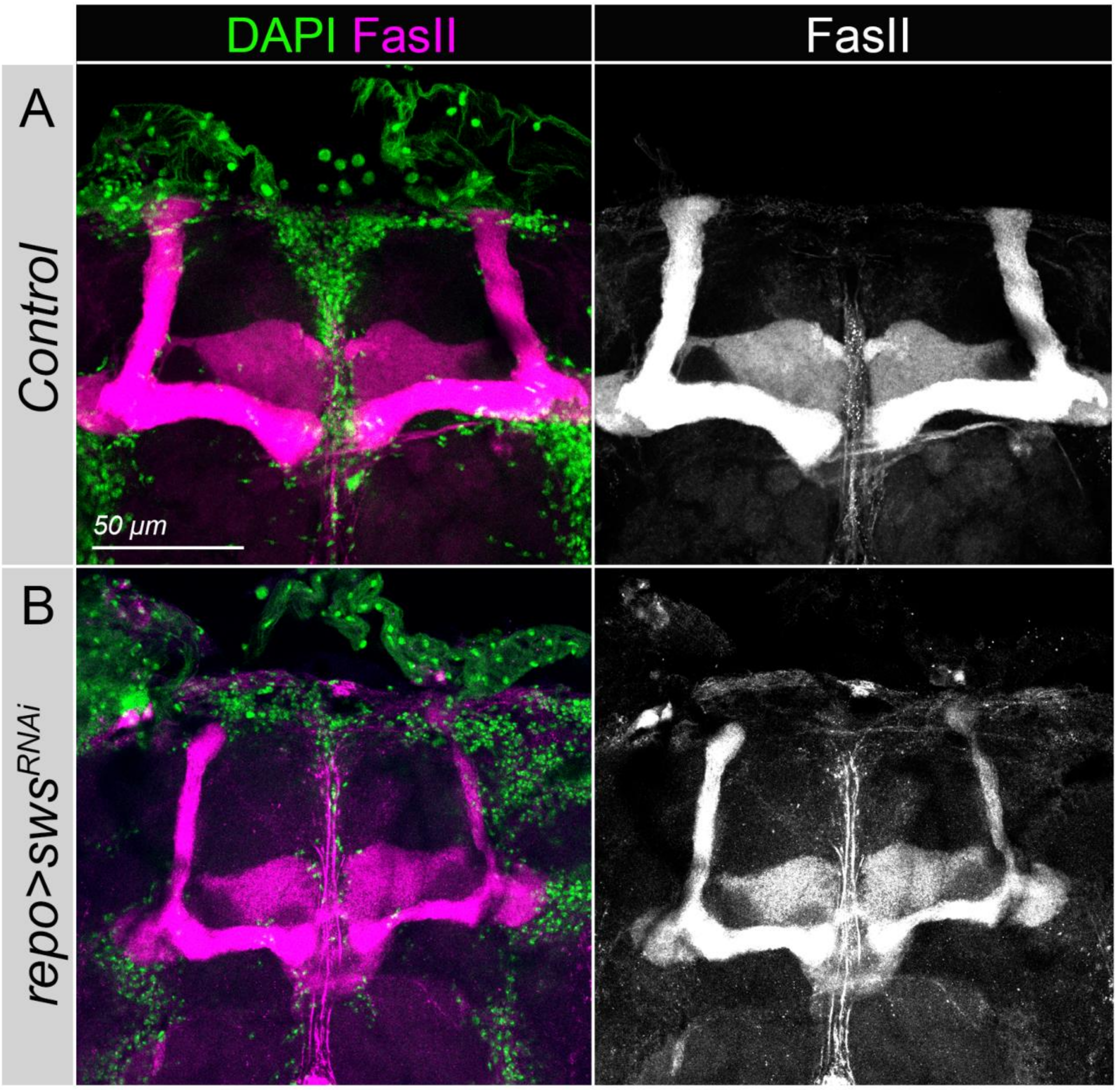

Figure S2. Downregulation of *sws* in glia (*repo-GAL4;UAS-sws-RNAi*) results in the mushroom body defects. (**A**) Confocal images of adult MB lobes in control flies (*repo-GAL4*/+) marked by FasII antibodies (magenta). (**B**) Upon *sws* downregulation in glia (*repo-GAL4;UAS-sws-RNAi*), MB lobes appear to be reduced.

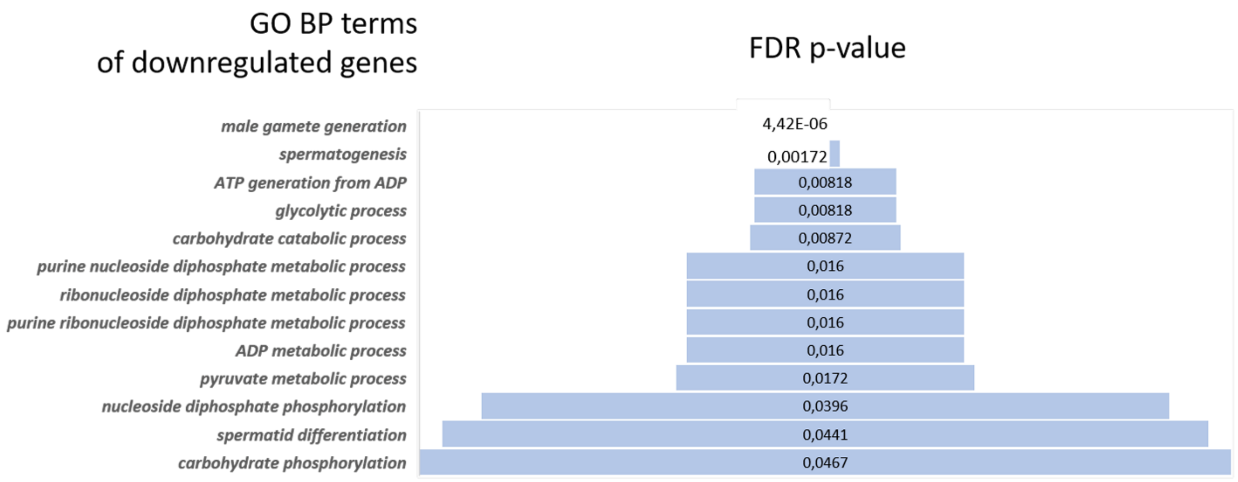

Figure S3. GO BP processes under the control of downregulated genes in 25-day-old neuronal *sws* knockdown males (*elav-GAL4;UAS-sws-RNAi*) compared to *CantonS* control and respective FDR-adjusted p-values of functional enrichment analysis in g:Profiler software.

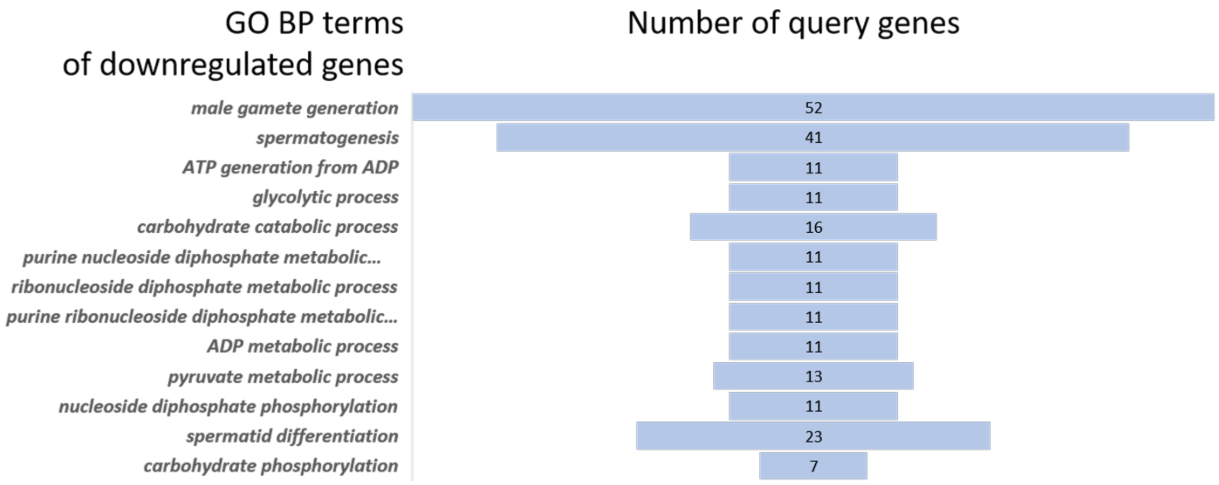

Figure S4. GO BP processes under the control of downregulated genes in 25-day-old neuronal *sws* knockdown males (*elav-GAL4;UAS-sws-RNAi*) compared to *CantonS* control and respective query gene number from functional enrichment analysis in g:Profiler software.

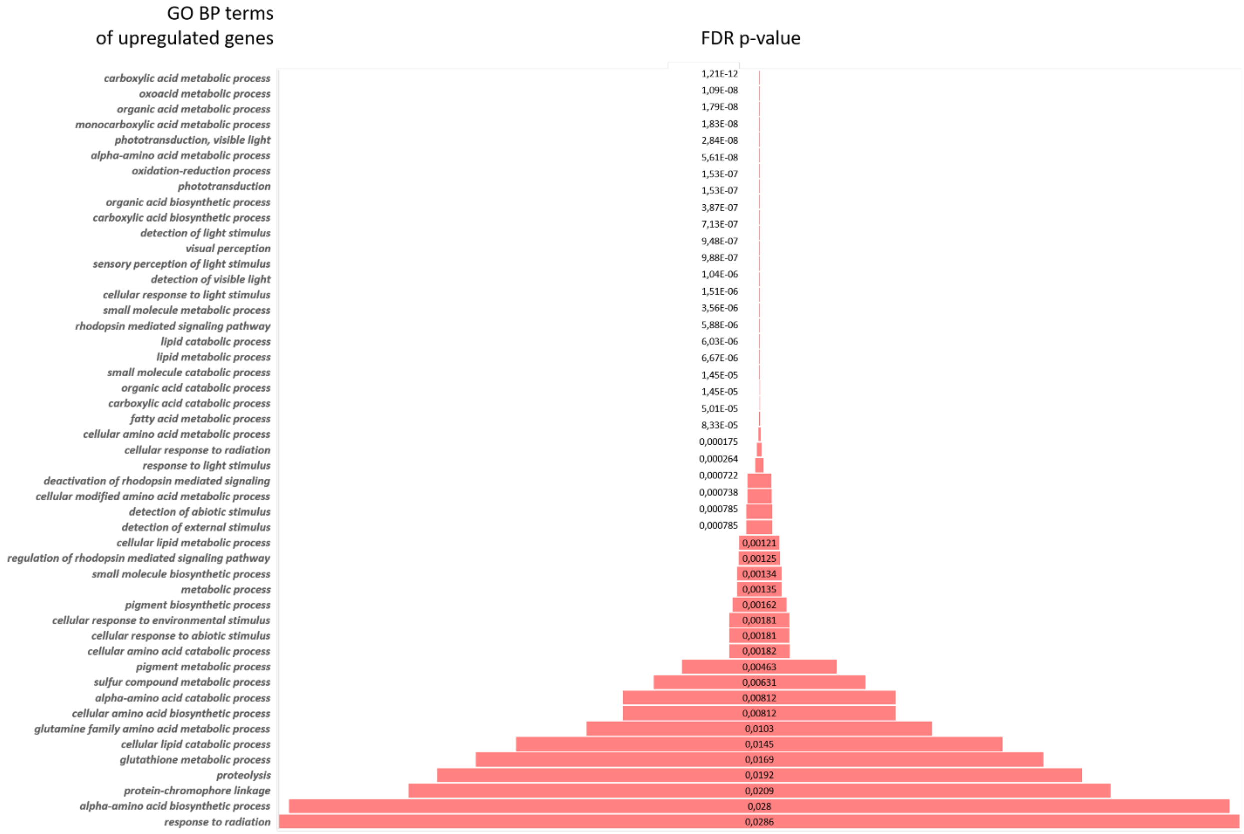

Figure S5. GO BP processes under the control of upregulated genes in 25-day-old neuronal *sws* knockdown males (*elav-GAL4;UAS-sws-RNAi*) compared to *CantonS* control and respective FDR-adjusted p-values of functional enrichment analysis in g:Profiler software.

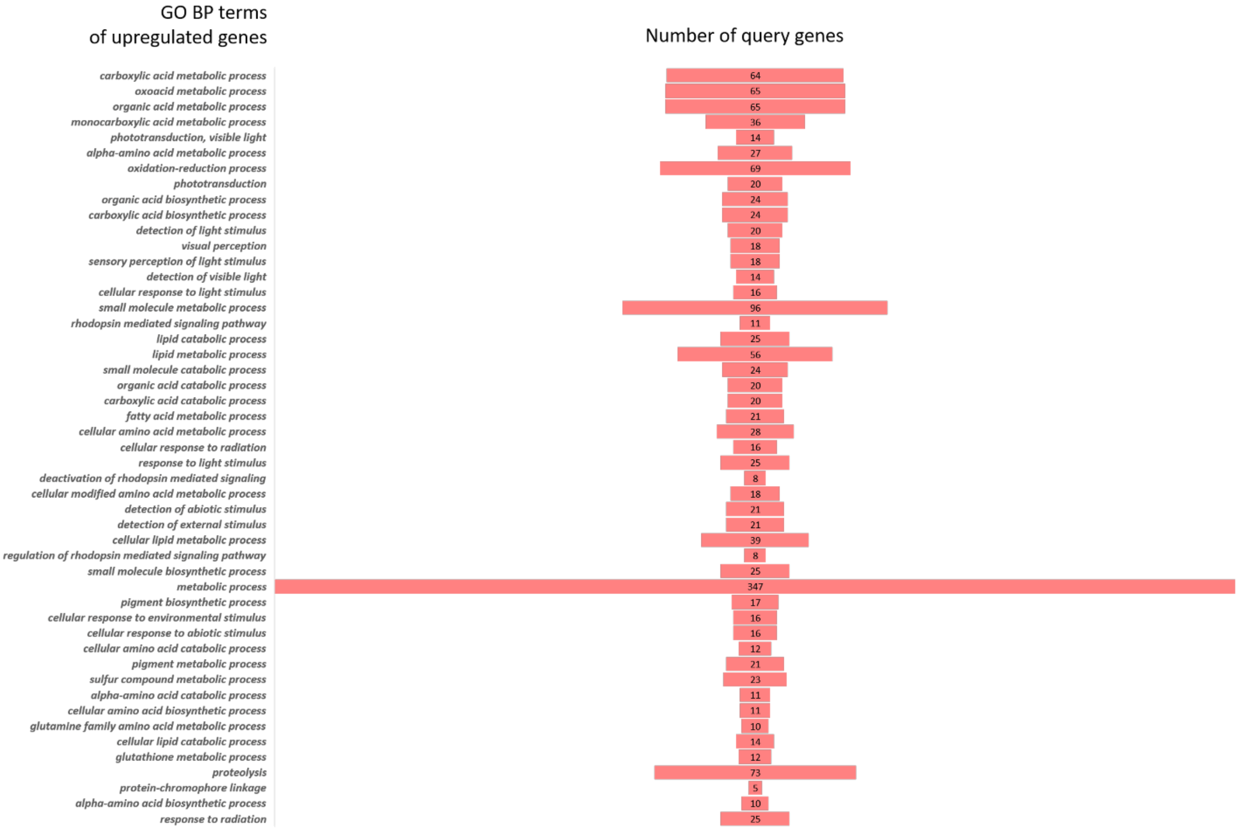

Figure S6. GO BP processes under the control of upregulated genes in 25-day-old neuronal *sws* knockdown males (*elav-GAL4;UAS-sws-RNAi*) compared to *CantonS* control and respective query gene number from functional enrichment analysis in g:Profiler software.

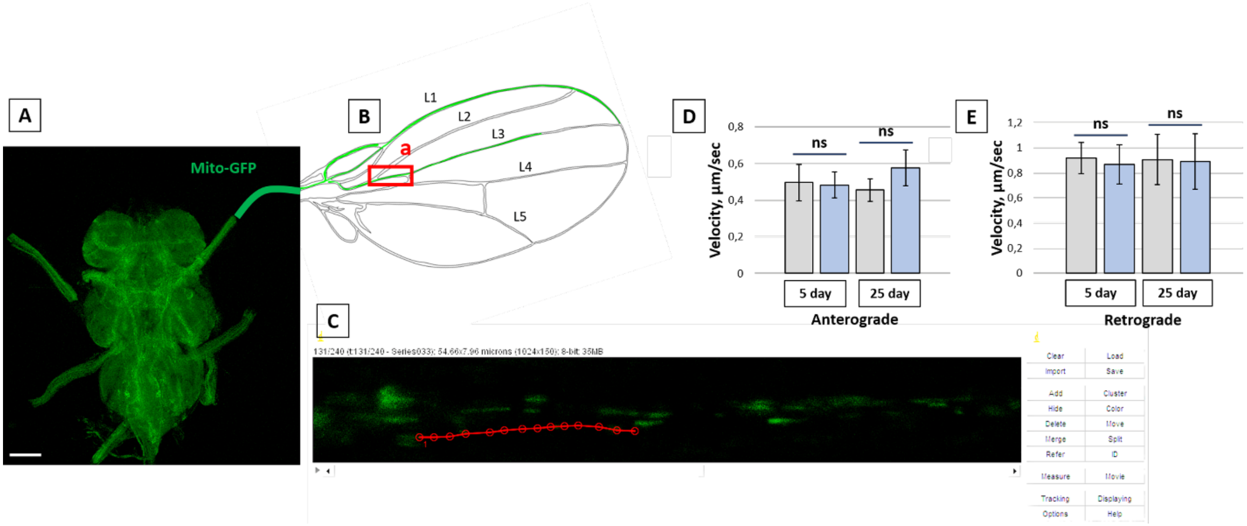

Figure S7. Analysis of axonal transport in the wing neuron. (**A**) 3-dimenshional confocal images of a ventral nerve cord of the 5-day-old control (*elav-GAL4;UAS-mito-GFP/+*) flies where GFP (green) is localised in mitochondria. Scale bar: 50 µm. (**B**) *Drosophila* wing fly structure scheme, (**a**, red region) – the area for mitochondrial transport velocity measurements. (**C**) The tracking for calculating mitochondrial transport velocity using ImageJ (MTrackJ plugin). (**D**) Anterograde and (**E**) retrograde mitochondria transport velocity (µm/sec) in the wing axons of the 5-day-old and 25-day-old control (*elav-GAL4;UAS-mito-GFP/+*) and *sws* knockdown (*elav-GAL4;UAS-mito-GFP/UAS-sws-RNAi*) flies. Mean ± 95% CI, Student t-test, ns – no significant difference ($p > 0.05$), $N = 25$.

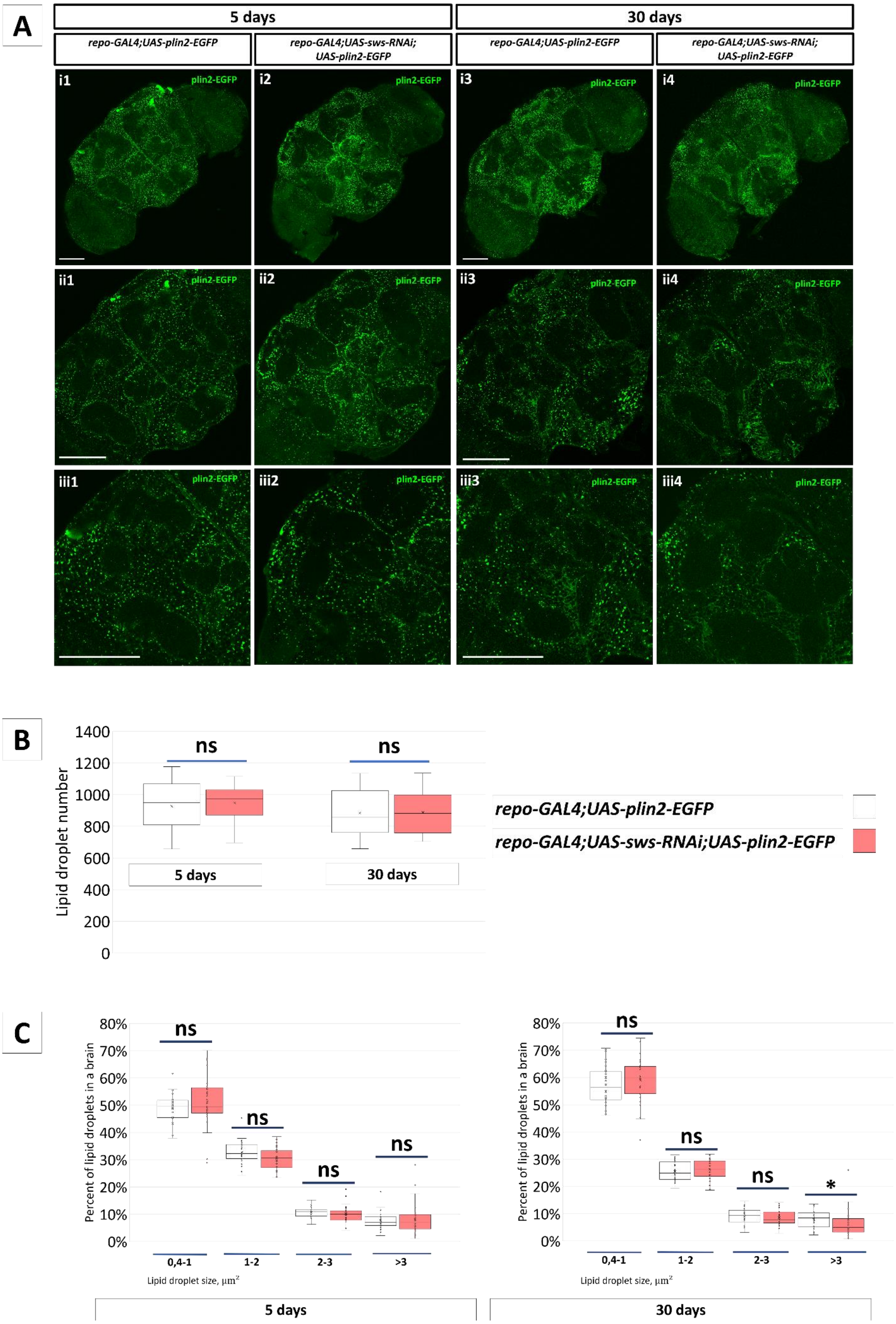

Figure S8. Analysis of lipid droplets in glia of the fly brain. (**A**) Single confocal images of the central brain of the control (**i-iii1**, **i-iii3**, *repo-GAL4;UAS-plin2-EGFP*) and knockdown (**i-iii2**, **i-iii4**, *repo-GAL4;UAS-sws-*

RNAi;UAS-plin2-EGFP) flies of 5 (**i-iii1**, **i-iii2**) and 30-day-old (**i-iii3**, **i-iii4**) age. Green – GFP embedded in lipid droplets (marked with arrows) via the Plin2 protein in glia. Scale bar: 100 μm. (**B**) The total lipid droplet number in the brain glia of the control (*repo-GAL4;UAS-plin2-EGFP*, white boxes) and knockdown (*repo-GAL4;UAS-sws-RNAi;UAS-plin2-EGFP*, red boxes) flies of 5 and 30-day-old age. Mann-Whitney test, ns – no significant difference ($p > 0.05$), $N = 30$. (**C**) Distribution of LD size (μm^2) in the brain glia of the control (*repo-GAL4;UAS-plin2-EGFP*, white boxes) and knockdown (*repo-GAL4;UAS-sws-RNAi;UAS-plin2-EGFP*, red boxes) flies of 5 and 30-day-old age. Mann-Whitney test, *** $p < 0.001$, * $p < 0.05$, ns – no significant difference ($p > 0.05$), $N = 30$.
